# The impact of nutrition-specific interventions on nutritional knowledge, dietary intake, and anemia among lactating mothers in Bukavu, Democratic Republic of the Congo – a randomized controlled trial

**DOI:** 10.1186/s40795-025-01231-x

**Published:** 2026-01-13

**Authors:** Damaris Elisabeth Beitze, Céline Kavira Malengera, Theophile Barhwamire Kabesha, Jan Frank, Veronika Scherbaum

**Affiliations:** 1 Department of Food Biofunctionality (140b), Institute of Nutritional Sciences, University of Hohenheim, Stuttgart, 70599 Germany; 2 School of Medicine and Public Health, Université Evangélique en Afrique, Bukavu, Democratic Republic of the Congo; 3 Département de Nutrition, Centre de Recherche en Sciences Naturelles/Lwiro, D.S. Bukavu, Democratic Republic of the Congo; 4 Faculty of Medicine, Official University of Bukavu, Bukavu, Democratic Republic of the Congo

**Keywords:** Nutrition education, Lipid-based supplements, Nutritional knowledge, Dietary diversity, Anemia, Hemoglobin, Lactating mothers, DR Congo

## Abstract

**Background:**

Amelioration of maternal nutrition is crucial in the Democratic Republic of the Congo (DRC), that experiences political instability and malnutrition. This study aimed to evaluate the impact of nutrition-specific interventions on nutritional knowledge, dietary practices, and hemoglobin (Hb) status among lactating mothers in the Bukavu region, DRC.

**Methods:**

A randomized, controlled trial was conducted with 416 mother–infant pairs from 3–6 to 6–9 months postpartum. The mothers were classified according to their mid-upper-arm circumference (MUAC) and subsequently assigned to the following intervention and control groups by block randomization: low MUAC–nutrition education, low MUAC–lipid-based nutrient supplements, low MUAC–control; normal MUAC–control; high MUAC–nutrition education, high MUAC–control. The pre- and post-intervention assessments included questionnaires on nutritional knowledge and practice to calculate scores (range 0–1); 24 h dietary recalls to calculate the Dietary Diversity Score (DDS, range 0–10); and measurement of maternal Hb. Changes of these parameters from pre- to post-intervention were analyzed by linear mixed models.

**Results:**

The change of total knowledge score from pre- to post-intervention was significantly positively affected by reception of nutrition education with a model-based mean difference of 0.161 between mothers with nutrition education compared to those without (*p* = 0.000). The change in DDS was not affected, but there was a positive impact of nutrition education on the modification of complementary feeding practices (OR 2.930, *p* = 0.030). Change in maternal Hb was not different between the intervention and control groups with higher increases among mothers with anemia compared to all mothers.

**Conclusion:**

Nutrition education can positively influence knowledge and potentially complementary feeding, while further measures may be required for beneficial effects on anemia.

**Trial registration:**

The study was registered prospectively at the German Clinical Trials Register (DRKS; DRKS-ID DRKS00012842) on November 27, 2017.

**Supplementary Information:**

The online version contains supplementary material available at 10.1186/s40795-025-01231-x.

## Background

The Democratic Republic of the Congo (DRC) and its eastern province South Kivu experience political instability and violence [1, 2] and high rates of poverty [3], that impair the availability of and access to food. Thus, the country suffers from high rates of food insecurity (69.2% in 2018–2020 [4]) and poor nutritional status (14.4% underweight and 38.4% anemia among women aged 15–49 years in 2013–14 [5]). According to the FAO, food security is based on the four pillars availability, access, utilization, and stability over time [6]. Previous studies reported associations of food security with dietary diversity [7–10] and affordability as one important driver of food choice, among others such as availability, health issues, or taste [11–13].

Deficiencies of iron and vitamin A are common in developing countries, however, various micronutrient deficiencies often coexist [14–17]. During pregnancy and lactation, nutrient requirements are elevated [18], making mothers vulnerable to malnutrition. Furthermore, mothers are mainly responsible for food preparation and, thus, family nutrition. Maternal dietary diversity was found to be associated with child dietary diversity [19, 20] and child anthropometrics [21].

Supplementation is effective in improving nutritional status [22–24]. Lipid-based nutrient supplements provide both a high energy and micronutrient content [25]. On the other hand, dietary diversity is associated with adequate nutrient supply [26–28]. To allow beneficial dietary choices, awareness about the utility of certain foods is crucial. Reception of nutrition information was found to be associated with higher dietary diversity [7, 29, 30]. Several authors emphasized that, besides declarative or factual knowledge (knowing nutritional facts), procedural or practical nutritional knowledge (knowing skills) is crucial for dietary behavior [31–35].

To the best of our knowledge, there is little information about the impact of factual and practical nutrition education covering both general nutritional knowledge and micronutrients on nutritional knowledge, practices, and anemia. Likewise, evidence about the impact of lipid-based nutrient supplements on anemia in lactating mothers is scarce. Especially in the context of ongoing conflict, the implementation and evaluation of nutrition-specific interventions is crucial. This study aimed to assess the impact of nutrition-specific interventions (lipid-based nutrient supplements, nutrition education) in under- and overweight lactating mothers in the DRC. We hypothesized that these interventions improve hemoglobin concentrations of the mothers. Secondary hypotheses included improved nutritional knowledge and dietary intake in mothers receiving nutrition education.

## Methods

This study is reported according to the CONSORT guidelines [36, 37].

### Study design

This multicenter, randomized controlled, parallel-group trial was embedded in a larger study with lactating mothers and their infants in Bukavu region, DRC. The study was conducted between December 2017 and June 2019 in two semi-urban areas and one rural area. Further details on the study setting are described elsewhere [38].

**Fig. 1:**
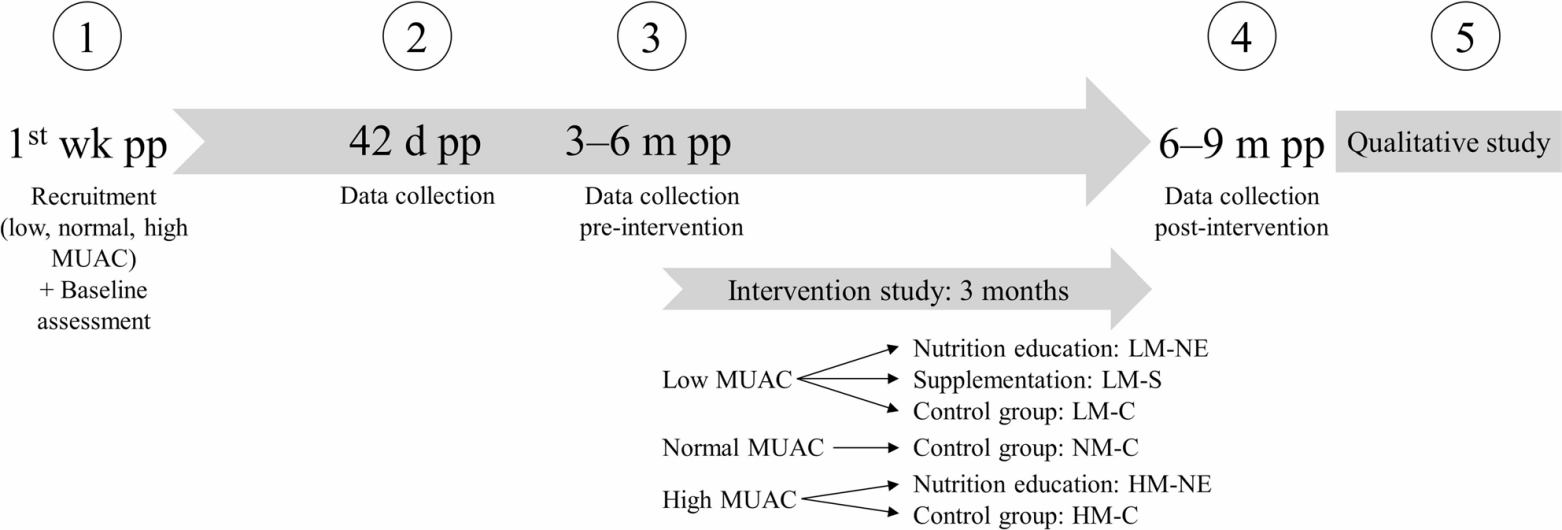
Study design. MUAC: mid-upper arm circumference, d: days, m: months, pp: postpartum, wk: week

The overall cross-sectional study with follow-up included four assessments for the mother–infant pairs, followed by a qualitative study with a subgroup of mothers (Fig. 1). The time points of the first four assessments were routine check-ups at the hospitals (first and second) and related health centers (third and fourth). Between the third and fourth assessment (3–6 and 6–9 months postpartum), the randomized controlled trial was conducted. This work presents the study period from the third to fourth assessment for evaluating the impact of the interventions. Results on nutritional knowledge, attitudes, practices, and anemia at the third assessment (pre-intervention) were presented in detail in a previous publication [39]. Data collection at these two assessments (pre- and post-intervention) included maternal mid-upper arm circumference (MUAC), nutritional knowledge and practice, dietary intake, and hemoglobin concentration (Hb).

At pre-intervention (third assessment), mothers were classified according to their MUAC and subsequently randomly allocated to intervention and control groups. To achieve a sufficient sample size for the intervention study, each eligible mother with low MUAC (≥ 21 and < 25 cm) and high MUAC (≥ 28 cm) and every third mother with normal MUAC (≥ 25 and < 28 cm) had been recruited (first assessment) [38]. As the intervention trial started only at the third assessment according to study design and changes in maternal MUAC compared to first assessment were noticed, the allocation into MUAC classes and subsequent intervention and control groups was based on their MUAC at the third assessment. Thereby, group sizes differed from the original calculated ones.

All study activities (data collection, provision of interventions) were performed in Swahili by trained local health personnel.

### Study participants

Inclusion and exclusion criteria are described in detail elsewhere [38, 39]. In brief, mothers were eligible for participation together with their newborn when aged 18–45 years, not severely underweight, and delivering a single healthy, full-term newborn in one of three study hospitals. Mothers or infants who developed severe underweight and/or anemia were referred to hospital for treatment but remained in the study. Reported treatments were reception of iron + folic acid syrup. Recruitment took place within the first week postpartum on condition of written informed consent. As this manuscript focuses on the mothers, results on the infants, including infant feeding practices, will be presented elsewhere.

### Interventions

Within the MUAC classes, mothers were randomly assigned to the intervention and control groups shown in Fig. 1. These groups were: low MUAC–nutrition education (LM-NE), low MUAC–supplement (LM-S), low MUAC–control (LM-C), normal MUAC–control (NM-C), high MUAC–nutrition education (HM-NE), high MUAC–control (HM-C). The intervention period started after the third assessment and lasted three months. The NM-C group was included for the purpose of the larger study observing mothers of all MUAC classes and their infants despite the lack of an intervention group of mothers with normal MUAC.

Nutrition education was specifically developed for this study based on a previous evaluation of local nutritional concepts and literature on frequent nutritional problems in developing countries that include iron and vitamin A deficiency [14, 15]. Locally, the three food group model is taught (food of energy, construction, protection) [40], thus, the study education was based on this concept. It consisted of four theoretical counseling sessions about balanced nutrition (three food group model), iron and anemia, vitamin A and its deficiency, and malnutrition, complemented by two cooking sessions on the preparation of nutrient-dense porridge and small pancakes. The cooking sessions related to the content of the theoretical sessions (three food group model, iron- and vitamin A-rich ingredients). The recipes aimed to enhance the nutritional quality of typical local foods following the concept of food-to-food fortification [41]. To realize an approach that is affordable for the study participants, they were plant-based with a mixture of cereal or manioc flour with powdered groundnuts, soy or insect flour in varying combinations, enriched by either fruits or vegetables. The principle of fermentation or roasting of the flour mix was explained and the importance of a thick porridge emphasized. All educational sessions were conducted by means of illustrated counseling cards with a participatory approach in a bi- to four-weekly rhythm. All education providers had received the same education material and advice about provision.

Mothers in the supplement group (LM-S) received the lipid-based nutrient supplement Plumpy’Mum™ by Nutriset (Malaunay, France) daily for three months. They were provided in a biweekly rhythm. One daily portion (92 g) contained 515 kcal and 35 mg iron, besides other micronutrients (see supplementary Table S1 for nutritional values).

Reception of any intervention was monitored by recording the dates of reception and potential self-reported leftovers of the supplements in the lists of participants. In case of single missed education sessions or supplement leftovers, mothers were still included into analysis, providing results of the interventions’ performance under field conditions.

Mother–infant pairs in the control groups did not receive any intervention during the project period.

All study participants received usual care, consisting of the routine check-ups with health counseling as a group and vaccinations at the health centers. This study did not assess if further campaigns provided health care or advice in the villages.

### Outcomes

The primary indicator was maternal hemoglobin concentration (Hb). Secondary indicators were maternal nutritional knowledge and practices and dietary intake. All of them were assessed at pre- and post-intervention (third and fourth assessment).

Data collection and processing were described in detail elsewhere [39]. In brief, Hb concentration was measured in blood gained by finger prick using the HemoCue Hb 201+ system (HemoCue AB, Ängelholm, Sweden) on site and adjusted to altitude by −0.5 g/dL [42]. Mild, moderate, and severe anemia were defined as follows: for non-pregnant mothers Hb = 11.0–11.9 g/dL, Hb = 8.0–10.9 g/dL, and Hb < 8.0 g/dL, respectively; for mothers reporting another pregnancy (*n* = 9 at post-intervention) Hb = 10.0–10.9 g/dL, Hb = 7.0–9.9 g/dL, and Hb < 7.0 g/dL, respectively [42, 43].

Maternal MUAC used for classification at pre-intervention was measured twice with a non-stretchable measuring tape (seca 212; seca GmbH & Co. KG, Hamburg, Germany) and mean was calculated. If the measures differed by more than 0.2 cm, they were repeated.

Nutritional knowledge and practice were assessed with a self-designed, pre-tested, structured questionnaire. It included 19 questions on knowledge and six questions on iron-related practices covering the topics provided by the nutrition education. Answers were rated as correct/wrong (knowledge) or beneficial/non-beneficial (practice). Knowledge and practice scores (K- and P-Scores) were calculated. They depict the proportion of correctly/beneficially answered questions out of all answered questions, ranging from 0 to 1. The following scores were calculated: K-Scores *three food group model*, *malnutrition*, *iron and anemia*, *vitamin A and vitamin A deficiency*, and *total* (including all knowledge questions); P-Score *iron*. Questions contributing to the scores are shown in supplementary Table S2.

At post-intervention, mothers were additionally asked for any changes in their own diet, breastfeeding behavior, or complementary food for the infant during the previous three months.

Maternal dietary intake was assessed by use of 24 h dietary recalls, conducted according to the 5-step multiple pass method of the United States Department of Agriculture [44]. Data were evaluated with EBISpro for Windows 2016 using the food composition table of the United States Department of Agriculture (SR 28) and African databases [45–47]. Food groups were allocated and the Dietary Diversity Score (range 0–10) and Minimum Dietary Diversity for Women (MDD-W, consumption of at least 5 out of 10 food groups) were calculated according to the FAO [26]. Mothers reporting any special occasions at the recall day were included into analysis if anything had been consumed (*n* = 8 and *n* = 9 at pre- and post-intervention) and excluded if no food had been consumed (*n* = 7 and *n* = 11).

### Sample size

The sample size calculation was based on the primary outcome of the difference of maternal Hb at post-intervention between intervention and control groups. Secondary outcomes were not included due to the focus on Hb as hard endpoint. Calculation was performed according to Allen, 2011 [48] with the following assumptions: Hb difference of Δ = 0.37 g/dL between the supplementation group and non-supplemented groups, standard deviation σ = 0.6 g/dL at post-intervention (adjusted to [49]), type 1 error probability at 0.05 (z_α/2_=1.96), statistical power at 90% (z_1-β_=1.28), assumed drop-out rate of 20%. The assumed drop-out rate was set at 20% to account for potential drop-outs from recruitment to the third assessment as well as during the intervention study. Calculated sample size was 420 (70 per group).

### Randomization and blinding

Mothers of low and high MUAC were assigned to the intervention and control groups by block randomization within each of the three study sites. Block randomization was used to achieve equal distribution of intervention and control groups within each study site. Sequence of random integers were generated by a true random number generator by the first author [50]. Blocks of 15 and 14 integers were used for low and high MUAC classes, respectively. Each block was sorted for the integers, intervention and control groups uniformly allocated (3 groups à 5 for low MUAC and 2 groups à 7 for high MUAC), and sorted back to original order. The resulting list was provided to the study team who assigned the mothers according to their order of appearance at the third assessment. Due to the nature of the interventions, blinding was not possible for neither study participants nor the study team.

### Statistical methods

Data analysis was performed using Microsoft Excel for Microsoft 365 MSO Version 2023, the Statistical Package for Social Sciences, version 27.0 (SPSS Inc., Chicago, IL, USA), and GraphPad Prism 8.0.1 for diagrams. Categorical data are expressed in *n* and %, and metric data in mean and standard deviation (SD), median and interquartile range (IQR). To account for the multi-center design, linear mixed models (LMMs) were computed for metric and generalized linear mixed models (GLMMs) with binary logistic regression for dichotomous dependent variables. For analyses regarding nutrition education, the intervention and control groups were combined as follows: Mothers receiving nutrition education (NE) as LM-NE + HM-NE, mothers with no nutrition education (NoNE) as LM-S + LM-C + NM-C + HM-C. This was based on the fact that both LM- and HM-mothers received the same nutrition education and supplementation or MUAC status were not expected to influence nutritional knowledge. Analyses on Hb and anemia compared all six intervention and control groups, while for DDS and MDD-W, both, comparison of NE and NoNE as well as all six groups was conducted.

The models were built with the following parameters. Dependent variables: change of Hb, DDS, and K-/P-Scores from pre-to post-intervention, MDD-W at post-intervention, and reported modification of maternal diet, breastfeeding, and complementary feeding. Fixed effects: intercept, intervention/control groups, the pre-intervention value of the dependent variable, hospitals (inter-hospital information was not used), and MUAC and the interaction of hospitals with MUAC for those analyses including the two groups NE and NoNE. Random effects: interaction of hospitals with intervention/control groups. All metric covariables were centered with grand-mean-centering before analysis. Interaction of hospital with MUAC was removed from the model if not significant. In case of non-convergence of the models, the random interaction was removed. If the model did not converge, hospital as fixed effect was removed from the model. Fixed and random effects of the final models are presented in supplementary Table S3. Satterthwaite approximation was used. Model-based means were calculated with pairwise comparisons. Normal distribution of residuals was assured by Q-Q-plots and variance homogeneity of residuals by plotting against the predicted values.

Values at pre- and post-intervention are presented descriptively. The changes from pre- to post-intervention are presented by use of the model-based means. Statistical significance was set at *P* < 0.05 (two-sided). Mother–infant pairs with incomplete datasets remained in the study with all information available. Sample size per variable/analysis is given.

## Results

**Fig. 2:**
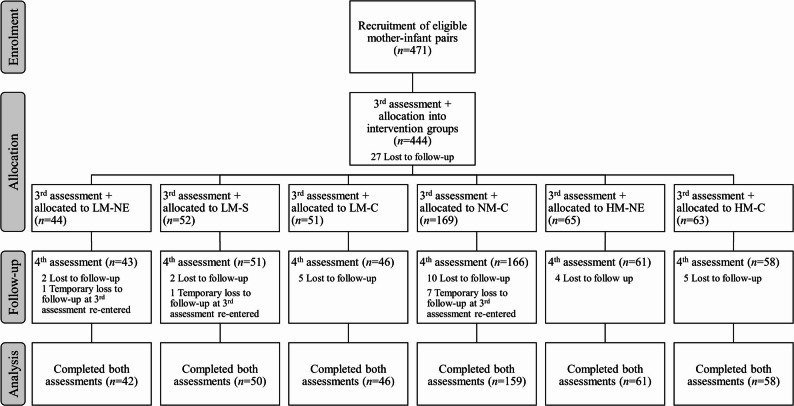
Flow diagram. LM-NE: low MUAC–nutrition education, LM-S: low MUAC–supplement, LM-C: low MUAC–control, NM-C: normal MUAC–control, HM-NE: high MUAC–nutrition education, HM-C: high MUAC–control

Baseline socio-demographic parameters (assessed from December 2017 to October 2018) are presented elsewhere [38]. At the third assessment (pre-intervention; March 2018 – January 2019), 444 mother–infant pairs still participated in the study; 425 pairs finished the study (post-intervention; June 2018 – April 2019). For 416 pairs, data are available pre- and post-intervention. They were included into analysis (Fig. 2). The sample includes mothers who reported termination of breastfeeding (*n* = 15 and *n* = 21 at pre- and post-intervention).

### Nutritional knowledge

**Fig. 3:**
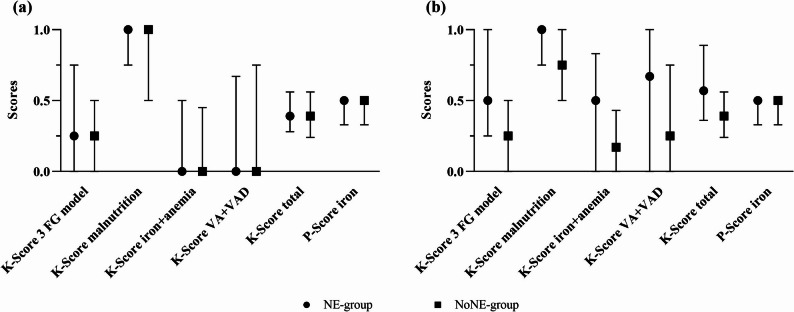
K- and P-Scores of lactating mothers at pre- and post-intervention; median, 25th and 75th percentile. **(a)** Pre-intervention (**b**) Post-intervention. 3 FG model: three food group model, K-Score: knowledge score, NE-group: nutrition education group (*n* = 103), NoNE-group: no nutrition education group (*n* = 313), P-Score: practice score, VA + VAD: vitamin A + vitamin A deficiency

 The descriptive analyses show similar knowledge in women assigned to NE- and NoNE-groups prior to the intervention, but higher post-intervention K-Scores among mothers in the NE-groups (Fig. 3, Table S4, S5). NE had a significant positive impact on all K-Score changes from pre- to post-intervention with a significantly higher mean change compared to NoNE-group, except for the K-Score *iron + anemia* (Table 1, S6, S7, S13). For all scores except K-Score *malnutrition*, the NE-group experienced a significantly positive change (Table 1). Descriptive analyses showed higher specific knowledge on foods belonging to the three food group model, iron- and vitamin A-rich foods, signs and symptoms of iron deficiency or vitamin A deficiency, and methods increasing iron bioavailability in the NE-group at post-intervention (Table S4).

**Table 1:** Estimated means of the change of K- and P-Scores from pre- to post-intervention according to the LMMs

	Mean	SE	95% CI		*P*-value
			Lower	Upper	
*K-Score three food group model (n = 408)* ^a^					
NE-group NoNE-group	0.237 0.056	0.034 0.022	0.170 0.013	0.305 0.100	
Mean difference NE - NoNE	0.181	0.037	0.108	0.254	0.000*
*K-Score malnutrition (n = 412)* ^a^					
NE-group NoNE-group	0.034 −0.053	0.033 0.021	−0.030 −0.095	0.098 −0.011	
Mean difference NE - NoNE	0.087	0.036	0.015	0.158	0.018*
*K-Score iron + anemia (n = 384)* ^a^					
NE-group NoNE-group	0.227 0.019	0.042 0.035	0.059 −0.363	0.395 0.401	
Mean difference NE - NoNE	0.209	0.054	−0.160	0.577	0.106
*K-Score vitamin A + vitamin A deficiency (n = 403)* ^a^					
NE-group NoNE-group	0.227 0.038	0.038 0.025	0.153 −0.011	0.300 0.086	
Mean difference NE - NoNE	0.189	0.041	0.108	0.270	0.000*
*K-Score total (n = 409)* ^a^					
NE-group NoNE-group	0.172 0.012	0.024 0.016	0.125 −0.019	0.220 0.043	
Mean difference NE - NoNE	0.161	0.027	0.108	0.213	0.000*
*P-Score iron (n = 397)* ^a^					
NE-group NoNE-group	0.055 0.004	0.015 0.010	0.025 −0.015	0.085 0.023	
Mean difference NE - NoNE	0.051	0.017	0.018	0.083	0.002*

*K-Score* Knowledge score, *NE* Nutrition education, *NoNE* No nutrition education; *P-Score* Practice score

*p < 0.05

^a^Lack of corresponding sum of frequencies with total sample size is due to missing data; total frequencies per variable are given

### Nutritional practice and dietary intake

The mean change of P-Score *iron* from pre- to post-intervention was significantly higher among NE- compared to NoNE-mothers with a significant effect of the intervention in the LMM (Table 1, S6, S7). Descriptive analyses showed similar practices at pre-intervention. At post-intervention, especially practice of fermentation and roasting of cereals/flour was still low but stated more often by mothers who participated in NE-sessions (Table S4).

**Fig. 4:**
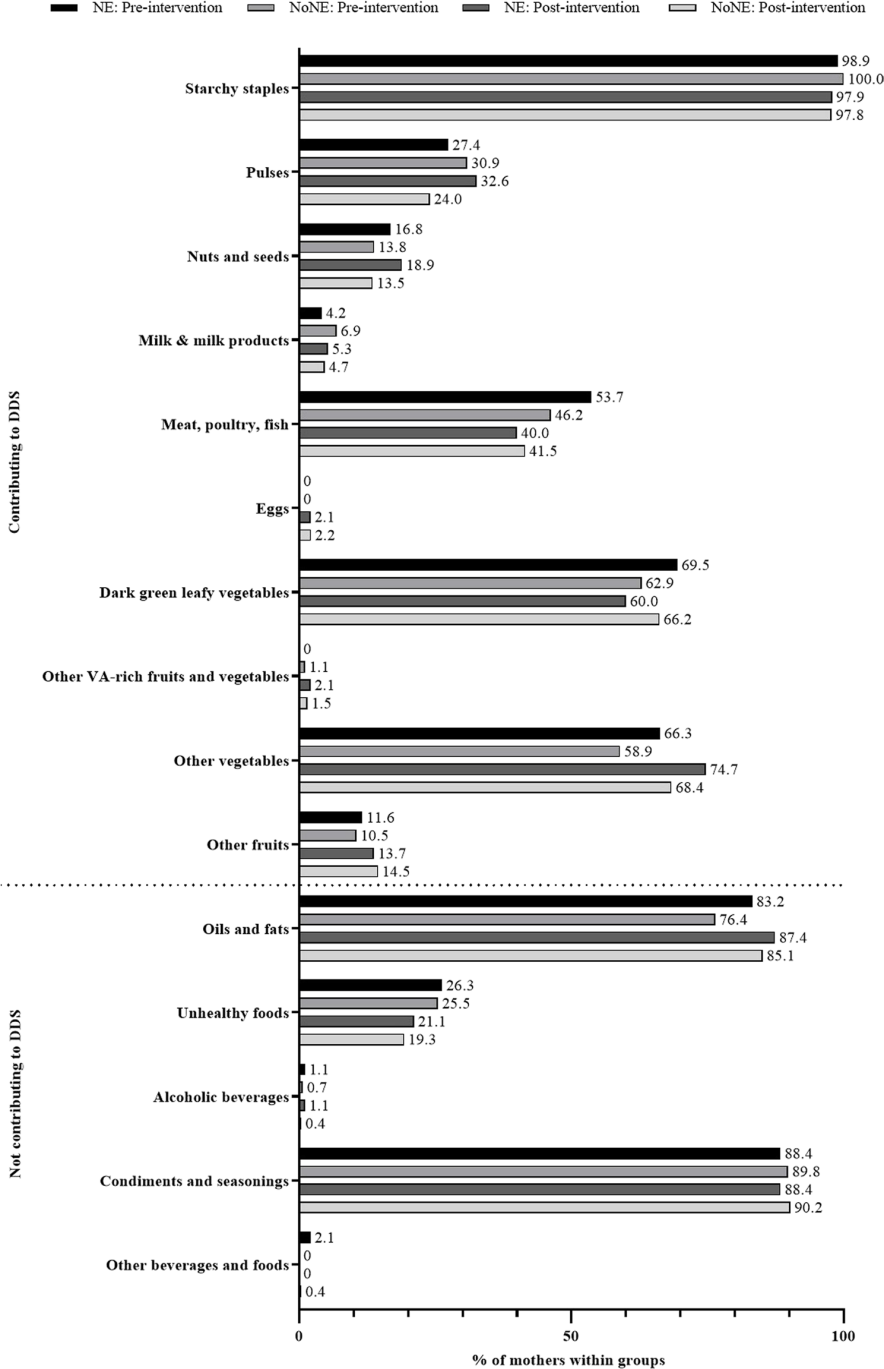
Consumption of food groups of lactating mothers at pre- and post-intervention. Frequencies are displayed as percentage. NE: nutrition education group (*n* = 95), NoNE: no nutrition education group (*n* = 275), VA: vitamin A

Descriptive analyses showed similar DDS and similar consumption of food groups at pre- and post-intervention among mothers in the NE- and NoNE-group (Table 2; Fig. 4) and slight differences between the six intervention and control groups (Table S14).There was no significant effect of the interventions on the change of DDS from pre-to post-intervention, neither in comparison of mothers receiving and not-receiving NE nor between all six intervention and control groups (Table 3, S6, S8, S9, S13). Equally, the odds ratio for the effect of NE- compared to NoNE-group on the MDD-W at post-intervention was not significant (OR 0.533; 95% CI: 0.229, 1.239), neither were odds ratios comparing the six intervention and control groups with NM-C as reference group (Table S6, S8, S13).

**Table 2:** Dietary diversity of mothers in nutrition education group and no nutrition education group at pre- and post-intervention

Variables^a^	Nutrition education *n*= 95		No nutrition education *n*= 275		Total^b ^*N*= 370	
*Dietary diversity score*						
	Mean Median	SD (IQR)	Mean Median	SD (IQR)	Mean Median	SD (IQR)
DDS pre-intervention	3.5 3.0	1.2 (3.0, 4.0)	3.3 3.0	1.1 (3.0, 4.0)	3.4 3.0	1.1 (3.0, 4.0)
DDS post-intervention	3.5 4.0	1.0 (3.0, 4.0)	3.3 3.0	1.1 (3.0, 4.0)	3.4 3.0	1.0 (3.0, 4.0)
*Minimum dietary diversity for women*						
	*n*	%	*n*	%	*n*	%
MDD-W pre-intervention						
≥ 5 food groups < 5 food groups	19 76	20.0 80.0	39 236	14.2 85.8	58 312	15.7 84.3
MDD-W post-intervention						
≥ 5 food groups < 5 food groups	11 84	11.6 88.4	38 237	13.8 86.2	49 321	13.2 86.8

*DDS* Dietary Diversity Score, *MDD-W* Minimum Dietary Diversity for Women

^a^Metric variables are expressed as mean ± SD and median (IQR), categorical variables as *n* and %

^b^Lack of corresponding sum of frequencies with total sample size is due to missing data; total frequencies per variable are given

**Table 3:** Estimated means of the change of DDS from pre- to post-intervention according to the LMMs

	Mean	SE	95% CI		*P*-value
			Lower	Upper	
*DDS with NE vs. NoNE (n = 369)* ^a^					
NE-group	0.236	0.131	−0.225	0.698	
NoNE-group	0.225	0.103	−0.918	1.369	
Mean difference NE - NoNE	0.011	0.162	−1.059	1.081	0.955
*DDS with six intervention and control groups (n = 370)* ^a, b^					
LM-NE	0.152	0.164	−0.171	0.475	
LM-S	0.414	0.161	0.096	0.731	
LM-C	0.108	0.167	−0.220	0.437	
NM-C	0.135	0.096	−0.055	0.325	
HM-NE	0.323	0.137	0.053	0.592	
HM-C	0.284	0.141	0.006	0.562	

*DDS* Dietary Diversity Score, *HM-C* High MUAC–control, *HM-NE* High MUAC–nutrition education, *LM-C* Low MUAC–control, *LM-NE* Low MUAC–nutrition education, *LM-S* Low MUAC–supplement, *NE* Nutrition education, *NM-C* Normal MUAC–control, *NoNE* No nutrition education

^a^Lack of corresponding sum of frequencies with total sample size is due to missing data; total frequencies per variable are given

^b^No significant mean difference between any of the groups. Pairwise comparisons are presented in Table S9

At post-intervention, mothers in the NE-group had a significantly higher probability of a modification of the complementary food they prepared for the child in the previous three months (OR: 2.930; 95% CI: 1.109, 7.742) while there were no significant effects on the modification of their own diet (OR: 1.534; 95% CI: 0.721, 3.263) or breastfeeding behavior (OR: 0.699; 95% CI: 0.238, 2.052) (Table 4, S6, S10, S15–S17). For complementary foods, NE-mothers mainly reported practices related to the content of the nutrition education such as addition of fruits or vegetables to the porridge or porridge consistency. Five out of eleven mothers justified their new behavior by the study education, and two related it to the iron or vitamin A content of the food (Table S17).

**Table 4:** Self-reported modification of dietary behavior of mothers in nutrition education group and no nutrition education group at post-intervention

Variables^a^	Nutrition education * n* = 103		No nutrition educaction * n* = 313		Total^b^ * N* = 416	
	*n*	%	*n*	%	*n*	%
Modification of own diet	*n* = 103		*n* = 311		*n* = 414	
Yes No Do not know	15 88 0	14.6 85.4 0.0	30 280 1	9.6 90.0 0.3	45 368 1	10.9 88.9 0.2
Modification of breastfeeding behavior	*n* = 101		*n* = 308		*n* = 409	
Yes No	9 92	8.9 91.1	23 285	7.5 92.5	32 377	7.8 92.2
Modification of complementary feeding	*n* = 103		*n* = 313		*n* = 416	
Yes No	11 92	10.7 89.3	11 302	3.5 96.5	22 394	5.3 94.7

^a^Categorical variables are expressed as % (n)

^b^Lack of corresponding sum of frequencies with total sample size is due to missing data; total frequencies per variable are given

### Hemoglobin and anemia

**Fig. 5:**
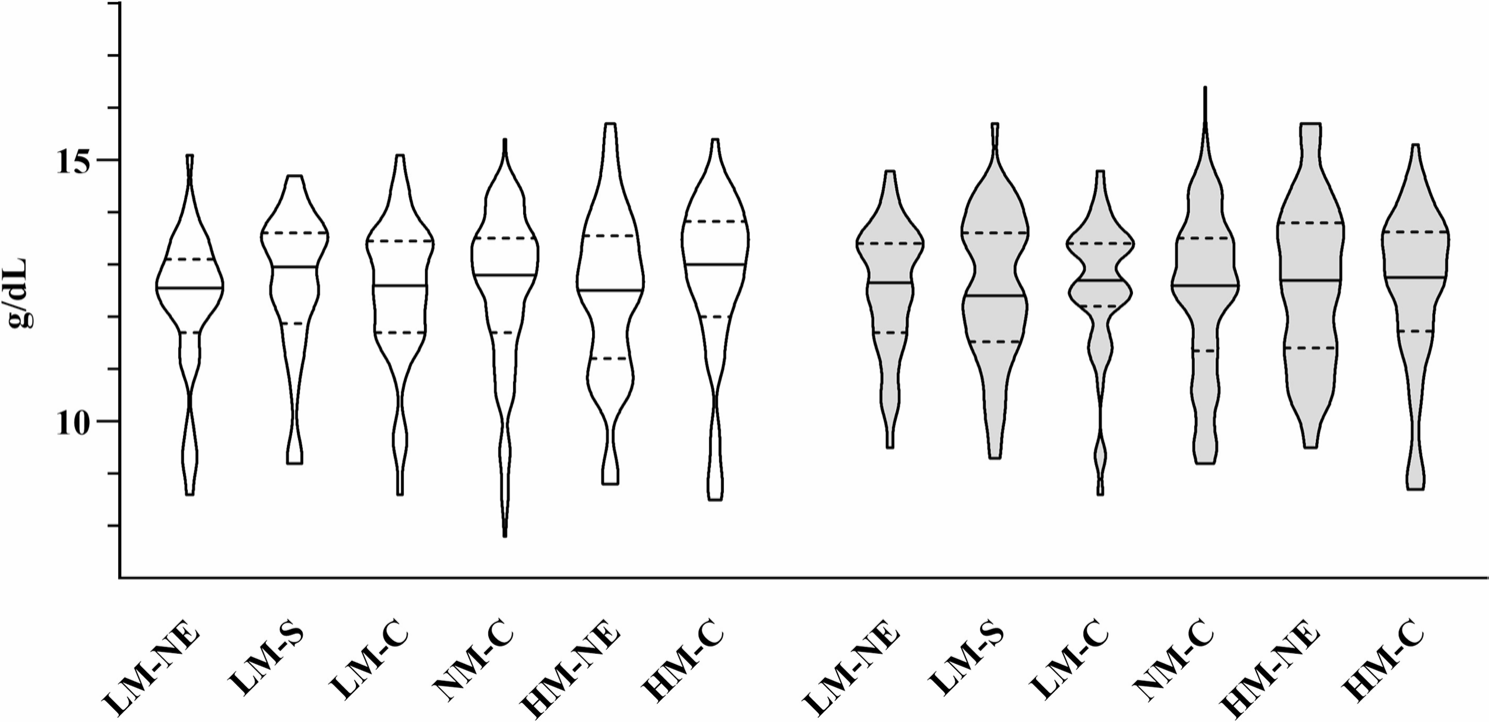
Hemoglobin concentration at pre- and post-intervention. Lines representing median, dotted lines 25th and 75th percentile. LM-NE: low MUAC–nutrition education (*n* = 40), LM-S: low MUAC–supplement (*n* = 48), LM-C: low MUAC–control (*n* = 45), NM-C: normal MUAC–control (*n* = 154), HM-NE: high MUAC–nutrition education (*n* = 58), HM-C: high MUAC–control (*n* = 54)

Descriptive analyses showed similar Hb-concentrations among the mothers in the six intervention and control groups at pre- and post-intervention and no improvements of anemia rates in the intervention groups (Fig. 5; Table 5). There was no significant impact of any of the interventions on the change of maternal Hb from pre- to post-intervention and no significantly different mean changes between the intervention and control groups (Table 6, S6, S11, S12). In separate analysis of only mothers with anemia at pre-intervention, there was no impact of intervention groups found, either, but the mean Hb changes were higher in all groups compared to the whole sample (Table 6S6, S11-S13).

**Table 5:** Hemoglobin concentration and anemia in mothers at pre- and post-intervention

Variables ^a^	Low MUAC – nutrition education	Low MUAC – supplement	Low MUAC – control	Normal MUAC – control	High MUAC – nutrition education	High MUAC – control	Total
***All mothers***	***n*** ** = 40**	***n*** ** = 48**	***n*** ** = 45**	***n*** ** = 154**	***n*** ** = 58**	***n*** ** = 54**	***N*** ** = 399**
Hemoglobin (g/dL)							
Pre-intervention	12.3 ± 1.4 12.6 (11.7, 13.1)	12.6 ± 1.4 13.0 (11.9, 13.6)	12.5 ± 1.4 12.6 (11.7, 13.5)	12.5 ± 1.5 12.8 (11.7, 13.5)	12.4 ± 1.6 12.5 (11.2, 13.6)	12.7 ± 1.6 13.0 (12.0, 13.8)	12.5 ± 1.5 12.7 (11.7, 13.5)
Post-intervention	12.5 ± 1.2 12.7 (11.7, 13.4)	12.5 ± 1.4 12.4 (11.5, 13.6)	12.6 ± 1.3 12.7 (12.2, 13.4)	12.4 ± 1.6 12.6 (11.4, 13.5)	12.6 ± 1.6 12.7 (11.4, 13.8)	12.5 ± 1.6 12.8 (11.7, 13.7)	12.5 ± 1.5 12.6 (11.5, 13.5)
Anemia pre-intervention							
No Mild Moderate Severe	75.0 (30) 10.0 (4) 15.0 (6) 0.0 (0)	75.0 (36) 12.5 (6) 12.5 (6) 0.0 (0)	68.9 (31) 20.0 (9) 11.1 (5) 0.0 (0)	72.1 (111) 12.3 (19) 14.9 (23) 0.6 (1)	65.5 (38) 12.1 (7) 22.4 (13) 0.0 (0)	77.8 (42) 11.1 (6) 11.1 (6) 0.0 (0)	72.2 (288) 12.8 (51) 14.8 (59) 0.3 (1)
Anemia post-intervention							
No Mild Moderate	70.0 (28) 17.5 (7) 12.5 (5)	64.6 (31) 20.8 (10) 14.6 (7)	77.8 (35) 13.3 (6) 8.9 (4)	68.8 (106) 9.7 (15) 21.4 (33)	63.8 (37) 20.7 (12) 15.5 (9)	70.4 (38) 14.8 (8) 14.8 (8)	68.9 (275) 14.5 (58) 16.5 (66)
***Mothers with anemia at pre-intervention***	***n*** ** = 10**	***n*** ** = 12**	***n*** ** = 14**	***n*** ** = 43**	***n*** ** = 20**	***n*** ** = 12**	***N*** ** = 111**
Hemoglobin (g/dL)							
Pre-intervention	10.3 ± 1.1 10.4 (9.3, 11.3)	10.7 ± 1.0 11.1 (9.4, 11.7)	10.9 ± 1.0 11.5 (9.8, 11.7)	10.5 ± 1.1 10.8 (9.6, 11.4)	10.6 ± 0.9 10.7 (10.5, 11.3)	10.2 ± 1.3 10.5 (9.0, 11.5)	10.5 ± 1.1 10.9 (9.6, 11.5)
Post-intervention	11.3 ± 1.3 11.0 (10.4, 12.6)	11.5 ± 1.2 11.7 (10.4, 12.3)	11.5 ± 1.5 11.9 (10.8, 12.5)	11.2 ± 1.5 11.2 (9.8, 12.2)	11.6 ± 1.2 11.4 (10.6, 12.6)	11.3 ± 1.4 11.4 (10.4, 12.4)	11.4 ± 1.4 11.4 (10.4, 12.4)

^a^Metric variables are expressed as mean ± SD and median (IQR), categorical variables as % (*n)*

^b^Lack of corresponding sum of frequencies with total sample size is due to missing data; total frequencies per variable are given

**Table 6:** Estimated means of the change of hemoglobin from pre- to post-intervention according to the LMMs

	Mean	SE	95% CI	
			Lower	Upper
***All mothers*** ***(n = 399)***^**a, b**^				
LM-NE	0.292	0.193	−0.089	0.672
LM-S	0.067	0.177	−0.281	0.416
LM-C	0.205	0.183	−0.156	0.565
NM-C	0.047	0.113	−0.176	0.269
HM-NE	0.267	0.163	−0.053	0.587
HM-C	−0.010	0.169	−0.342	−0.322
***Mothers with anemia at pre-intervention (n = 111)*** ^**a**^				
LM-NE	1.334	0.469	0.365	2.302
LM-S	1.327	0.440	0.396	2.259
LM-C	1.242	0.397	0.413	2.071
NM-C	1.021	0.273	0.352	1.690
HM-NE	1.313	0.333	0.599	2.027
HM-C	1.307	0.438	0.388	2.227

*HM-C* High MUAC–control, *HM-NE* High MUAC–nutrition education, *LM-C* Low MUAC–control, *LM-NE* Low MUAC–nutrition education, *LM-S* Low MUAC–supplement, *NM-C* Normal MUAC–control

*Significant effect

^a^Lack of corresponding sum of frequencies with total sample size is due to missing data; total frequencies per variable are given

^b^No significant mean difference between any of the groups. Pairwise comparisons are presented in Table S12

For all parameters except MDD-W and dietary modifications, the effect of the pre-intervention status was significantly negative in the respective LMM.

## Discussion

To the best of our knowledge, this is the first study that assessed the impact of a nutrition education intervention covering topics of both general nutrition and specific micronutrients on nutritional knowledge, practice, and anemia status. We found a positive impact of nutrition education on nutritional knowledge and iron-related practice, but not on dietary diversity.

The increased K- and P-Scores in the NE- compared to the NoNE-group are corroborated by previous studies that found improved knowledge and/or practices after differently designed nutrition education interventions [51–56]. Concerning effects on dietary intake, studies found improved women’s diets [52, 53, 57, 58] including DDS and nutrient intakes.

In this study, mothers receiving nutrition education showed more detailed knowledge at post-intervention, such as on foods belonging to the three food group model, iron- or vitamin A-rich foods, methods that increase iron bioavailability, or consequences of iron- or vitamin A deficiency. Thus, previously existing general knowledge was enriched with new information. Knowledge on symptoms of a deficiency may increase seeking of medical care in these occasions, while knowledge on foods and food processing might improve meal preparation. Higher rates of (maize) flour fermentation and roasting in NE-mothers indicate the implementation of taught processing methods. Regarding nutrient-rich foods or foods/methods influencing bioavailability, especially those were mentioned more frequently that had been used in the cooking sessions, supporting their important role. Altogether, both factual and procedural knowledge were increased.

Nevertheless, reasons for lack of translation of this knowledge into practice in terms of dietary diversity need to be elucidated. In accordance, at pre-intervention a limited relationship of knowledge and DDS was detected [39]. A qualitative study in Malawi revealed taste, lack of family support, as well as affordability and poverty as main barriers of implementation of messages from nutrition education [59]. In Rwanda and Ghana, no improvement of dietary intake despite increased knowledge after behavior change communication or nutrition education was linked to the sustained food environment and limited food availability [60, 61]. Similar drivers of food choice were reported by the mothers at pre-intervention [39], therefore, it is likely that affordability and personal preferences limited the implementation of knowledge. Around half of the mothers who had modified their own eating practices mentioned a reduced quantity, mostly due to financial constraints (Table S17). This supports the critical role of wealth and food prices that outweighs knowledge. Previously, it was found that education interventions should be combined with supplementation programs for a beneficial effect on children receiving complementary feeding in contexts of limited financial means [22]. On the other hand, some mothers in this study reported to have altered the complementary food they prepared for their infant as consequence of the education. Thus, knowledge might have been used in ways that were not reflected by their own DDS. This complies with previous findings of improved children’s diets after nutrition education for caretakers [51, 54]. The reported changes referred mainly to the contents of the practical education sessions with preparation of nutrient-rich porridge, thus emphasizing the importance of procedural knowledge. A review on behavior change interventions regarding mother and child nutrition found greater impacts of interventions with persuasion, incentivization, and environmental restructuring on dietary intake compared to education, training, and enablement [62]. Consequently, the combination of nutrition education with further measures is recommended to address the multifaceted drivers of food choice and enable behavior change. Multisectoral interventions, including direct and indirect actions, such as disease prevention, women’s empowerment, or poverty alleviation strategies are needed [63]. Combined cash transfers and digital nutrition education showed a positive effect on dietary diversity and food security in Sri Lanka [64], while evidence about the effect of multisectoral approaches on anemia is rare [65].

Some knowledge increase was found among NoNE-mothers, too. Similar results were reported by Katenga-Kaunda et al. [52]. We cannot exclude that some knowledge was transferred from NE- to NoNE-mothers as they were not spatially separated, or knowledge was acquired from other sources.

Considering all six intervention and control groups, no significant effect of any of them was found on the Hb change from pre- to post-intervention. The mean Hb change was highest in the NE-groups, though not statistically different from other groups. A meta-analysis found reduced anemia risk in pregnant women by nutrition education, especially when provided together with supplementation, and reported conflicting results regarding impact on Hb and iron status indicators [58]. A potential positive impact of nutrition education on Hb, but not DDS, is in line with the associations of Hb with K- and P-Scores, but not DDS, at pre-intervention [39].

Even among the mothers receiving supplements (LM-S), a meaningful mean Hb increase was only observed within those being anemic, though not significantly different from other groups. This finding underlines the targeted application in mothers with deteriorated micronutrient status rather than a general supplementation. To the best of our knowledge, there is limited evidence about the effect of lipid-based nutrient supplements in lactating women on Hb status or anemia. Among HIV-infected lactating women in Tanzania, lipid-based supplements hardly influenced Hb or anemia of the mother and infant, but could alleviate the adverse effect of antiretroviral treatment on maternal tissue iron stores [66]. However, the infantile inflammation-adjusted Hb was adversely affected.

The limited impact of both interventions on maternal Hb concentration might be caused by several factors. The intervention time was rather short, especially for a nutrition education program. Implementation of gained knowledge and subsequent recovery of Hb concentration needs time and may be affected by other determinants of dietary practices. Mothers receiving the supplements were advised to consume it completely on their own, however, some reported to have shared parts of it with their children. Thus, the ingested dose was partly lowered. Nevertheless, the slight increase of DDS in the LM-S group suggests that the supplement was correctly taken as addendum to the regular diet and no meals were replaced by it. In iron-repleted persons, iron absorption is downregulated via Hepcidin [67], thus, absorption of additional iron would be limited. This can explain the stronger Hb increase in anemic mothers receiving interventions compared to the whole groups including non-anemic mothers. Despite a positive development among mothers with anemia, anemia rates did not improve (Table 5). Mothers may have increased their Hb but still be in an anemic state. The median Hb concentrations of the mothers at pre-intervention were mostly to be classified as moderate anemia but improved to mild anemia at post-intervention. Furthermore, mothers who became anemic throughout the intervention phase may have masked those who recovered.

Nutrition education included information about a balanced nutrition in general, not only iron and anemia. Plumpy’Mum™ contains a wide variety of nutrients that were not measured in this study, neither were iron stores. Thus, other positive impacts might have occurred, that were not assessed in this study. Multi-nutrient deficiencies are likely to occur in the study area [14, 39], hence use of such multi-nutrient products and approaches is reasonable.

Maternal anemia was found in all MUAC-groups. Consequently, in anemia treatment, the selection of the supplement needs to be adjusted to the overall nutritional status. For underweight women, a lipid-based supplement like Plumpy’Mum™ is reasonable, for normal- or overweight anemic women, formulations with low energy content are more suitable. Nutrition education can be used irrespective of nutritional status.

For K- and P-Scores, DDS, and Hb, the status at pre-intervention was significant in the respective LMM with a negative direction. This underlines the greater Hb increases in mothers with anemia compared to all mothers. It further suggests targeted interventions according to a person’s status regarding nutritional knowledge, dietary practices, and nutritional status.

### Strengths and limitations

This study has several strengths. First, the impact of nutrition interventions on the whole chain of nutritional knowledge, practices, and Hb status was assessed. Nutrition education included general knowledge but also focused on specific nutrients. Lack of research focusing on such specific topics had been mourned previously [31]. Further, the theoretical and practical sessions considered the importance of including procedural knowledge rather than pure declarative knowledge [31] as well as local foods and processing methods [59]. Both assessments, before and after the intervention, included both rainy and dry seasons, thus, the results on dietary diversity are unlikely to be caused by seasonality.

It needs to be considered that, due to changes in MUAC of the mothers since the baseline assessments, the sample size within the intervention groups was reduced and fell below the initially calculated sample size per group, thus, the statistical power of several analyses was limited. A condensed study design with a lower number of intervention groups could overcome this limitation. This may include targeting interventions according to anemia- than MUAC-category due to the occurrence of anemia in all MUAC-groups. Assessing a single 24 h dietary recall per person at each assessment and reporting meals prepared by others with limited information about preparation may have led to some bias in DDS [26, 39]. In addition, social desirability may have led to an overestimation of the consumption of food groups that were considered to be beneficial. Similarly, potential bias in self-reported parameters may have occurred as blinding was not feasible. Lack of motivation in mothers of the control groups may have diminished the presentation of their knowledge. However, mothers were asked open questions, reducing the risk of false-positive answers in the intervention groups. The primary indicator, Hb concentration, is considered independent from the blinding. The intervention period was rather short, especially for a nutrition education program. It was chosen based on the capabilities of both the study participants and the study team. A longer duration and combination with approaches addressing availability and affordability of foods may be beneficial. Furthermore, we could only analyze the Hb concentration, but not nutrient status of the study participants. Conducting the study as a practical implication in the field caused limitations like delays in appearing to the appointments or limited intervention fidelity that could affect the impacts of interventions compared to optimal conditions. However, this approach shows the effects of an implementation of nutritional interventions under real field conditions.

## Conclusions

This study found a beneficial impact of nutrition education on nutritional knowledge. While dietary diversity was not improved, some changes in the complementary feeding practices were reported. Practical demonstration of preparation of beneficial foods should be an integral part of nutrition education strategies to provide procedural knowledge and address taste as one driver of food selection. There were only small and non-significant effects of either nutrition education or lipid-based nutrient supplements found on maternal hemoglobin concentration. Effects may have been limited by a short intervention duration and further factors influencing dietary intake and status. Greater Hb increases among mothers with anemia underline the importance of targeted interventions. Further research with a greater number of mothers with and without anemia is warranted to develop valuable designs of multisectoral interventions, combining nutrition education with further measures for targeted combating of malnutrition.

## Supplementary Information

Supplementary Material 1: Table S1. Nutritional values of the lipid-based supplement Plumpy’Mum™ (Nutriset). Table S2. Questions included in K- and P-Scores. Table S3. Fixed and random effects used in the (generalized) linear mixed models. Table S4. Nutritional knowledge and practices of mothers in nutrition education group and no nutrition education group at pre- and post-intervention. Table S5. Knowledge and practice scores of mothers in nutrition education group and no nutrition education group at pre- and post-intervention. Table S6. LMMs and GLMMs for the change of K- and P-Scores, DDS, and Hb of mothers from pre- to post-intervention, and MDD-W and dietary modifications at post-intervention. Table S7. Fixed coefficients of the LMMs for the change of K- and P-Scores from pre- to post-intervention. Table S8. Fixed coefficients of the LMMs and GLMMs for the change of DDS from pre- to post-intervention and MDD-W at post-intervention. Table S9. Estimated mean differences in the change of DDS from pre- to post-intervention between the six intervention and control groups according to the LMMs. Table S10. Fixed effects of the GLMMs for dietary modifications at post-intervention. Table S11. Fixed effects of the LMMs for the change of hemoglobin from pre- to post-intervention. Table S12. Estimated mean differences in the change of hemoglobin from pre- to post-intervention between the six intervention and control groups according to the LMMs. Table S13. Random effect covariance of the LMMs for the change of K- and P-Scores, DDS, and Hb of mothers from pre- to post-intervention. Table S14. Dietary diversity of mothers in the six intervention and control groups at pre- and post-intervention. Table S15. Self-reported modifications of own diet and reasons for modification by mothers in nutrition education group and no nutrition education group at post-intervention. Table S16. Self-reported modifications of breastfeeding behavior and reasons for modification by mothers in nutrition education group and no nutrition education group at post-intervention. Table S17. Self-reported modifications of complementary feeding and reasons for modification by mothers in nutrition education group and no nutrition education group at post-intervention.

## Data Availability

The datasets used during the current study are available from the corresponding author on reasonable request due to ongoing analysis and paper writing.

## Abbreviations

3 FG model: Three food group model
95% CI: 95% confidence interval
d: Days
DDS: Dietary Diversity Score
DRC: Democratic Republic of the Congo
GLMM: Generalized linear mixed model
Hb: Hemoglobin
HM-C: High MUAC–control
HM-NE: High MUAC–nutrition education
K-Score: Knowledge score
LMM: Linear mixed model
LM-C: Low MUAC–control
LM-NE: Low MUAC–nutrition education
LM-S: Low MUAC–supplement
m: Months
MDD-W: Minimum Dietary Diversity for Women
MUAC: Mid-upper-arm circumference
NE: Nutrition education
NM-C: Normal MUAC–control
NoNE: No nutrition education
OR: Odds ratio
pp: Postpartum
P-Score: Practice score
SE: Standard error
VA: Vitamin A
VAD: Vitamin A deficiency
wk: Week
