# Supplementary material for: The impact of nutrition-specific interventions on nutritional knowledge, dietary intake, and anemia among lactating mothers in Bukavu, Democratic Republic of the Congo – a randomized controlled trial
Source: BMC Nutr. 2026 Jan 13;12:15. doi: 10.1186/s40795-025-01231-x (PMC12853581; doi:10.1186/s40795-025-01231-x)
Supplement: Supplementary file 1 — Supplementary Material 1: Table S1. Nutritional values of the lipid-based supplement Plumpy’Mum™ (Nutriset). Table S2. Questions included in K- and P-Scores. Table S3. Fixed and random effects used in the (generalized) linear mixed models. Table S4. Nutritional knowledge and practices of mothers in nutrition education group and no nutrition education group at pre- and post-intervention. Table S5. Knowledge and practice scores of mothers in nutrition education group and no nutrition education group at pre- and post-intervention. Table S6. LMMs and GLMMs for the change of K- and P-Scores, DDS, and Hb of mothers from pre- to post-intervention, and MDD-W and dietary modifications at post-intervention. Table S7. Fixed coefficients of the LMMs for the change of K- and P-Scores from pre- to post-intervention. Table S8. Fixed coefficients of the LMMs and GLMMs for the change of DDS from pre- to post-intervention and MDD-W at post-intervention. Table S9. Estimated mean differences in the change of DDS from pre- to post-intervention between the six intervention and control groups according to the LMMs. Table S10. Fixed effects of the GLMMs for dietary modifications at post-intervention. Table S11. Fixed effects of the LMMs for the change of hemoglobin from pre- to post-intervention. Table S12. Estimated mean differences in the change of hemoglobin from pre- to post-intervention between the six intervention and control groups according to the LMMs. Table S13. Random effect covariance of the LMMs for the change of K- and P-Scores, DDS, and Hb of mothers from pre- to post-intervention. Table S14. Dietary diversity of mothers in the six intervention and control groups at pre- and post-intervention. Table S15. Self-reported modifications of own diet and reasons for modification by mothers in nutrition education group and no nutrition education group at post-intervention. Table S16. Self-reported modifications of breastfeeding behavior and reasons for modification by mothers in nut [file 40795_2025_1231_MOESM1_ESM.docx]

**Table S1.** Nutritional values of the lipid-based supplement Plumpy’Mum™ (Nutriset)

| **Nutrient** | **For 92 g (serving size)** |
| --- | --- |
| Energy | 515 kcal |
| Proteins | 14.4 g |
| Lipids | 36.6 g |
| Calcium | 111 mg |
| Zinc | 17 mg |
| Copper | 2.7 mg |
| Iron | 35 mg |
| Iodine | 150 μg |
| Selenium | 85 μg |
| Vitamin A | 881 μg* |
| Vitamin D | 15 μg |
| Vitamin E | 13 mg |
| Vitamin C | 71 mg |
| Vitamin B1 | 1.6 mg |
| Vitamin B2 | 1.6 mg |
| Vitamin B6 | 2 mg |
| Vitamin B12 | 2.6 μg |
| Folic acid | 461 μg |
| Niacin | 21 mg |

* 881 μg RE (retinol equivalents).

**Table S2.** Questions included in K- and P-Scores

| **K: *three food group model*** | **K: *malnutrition*** | **K: *iron + anemia*** | **K: *vitamin A + vitamin A deficiency (VAD)*** | **P: *iron*** |
| --- | --- | --- | --- | --- |
| Can state at least 1 of the 3 types of food | Can state a definition of malnutrition | Can state a sign/symptom of anemia | Can state a sign/symptom of VAD | Consumption of animal foods ≥ 1/wk |
| Can state an energy-giving food | Can state a consequence of malnutrition | Can state a consequence of iron deficiency in children |  | Consumption of green leafy vegetables ≥ 3x/wk |
| Can state a constructive food | Can state a cause of malnutrition | Can state a cause of anemia | Can state a cause of VAD | Consumption of fruits with a meal |
| Can state a protective food | Can state a preventive measure for malnutrition | Can state a preventive measure for anemia | Can state a preventive measure for VAD | Consumption of coffee/tea only outside a meal |
|  |  | Can state an iron-rich food | Can state a VA-rich food | Fermentation |
|  |  | Can state a method increasing iron bioavailability |  | Roasting of grains |
|  |  | Can state a drink reducing iron bioavailability |  |  |

K-Score: knowledge score, P-Score: practice score, VAD: vitamin A deficiency.

**Table S3.** Fixed and random effects used in the (generalized) linear mixed models

| **Dependent variable** | **Fixed effects^a^** | **Random effects** |
| --- | --- | --- |
| K-Score *three food group model* | Intervention NE vs NoNE  MUAC at pre-intervention  K-Score *three food group model* at pre-intervention  Hospital  Hospital x MUAC at pre-intervention | - |
| K-Score *malnutrition* | Intervention NE vs NoNE  MUAC at pre-intervention  K-Score *malnutrition* at pre-intervention  Hospital | - |
| K-Score *iron + anemia* | Intervention NE vs NoNE  MUAC at pre-intervention  K-Score *iron + anemia* at pre-intervention  Hospital | Hospital x Intervention NE vs NoNE |
| K-Score *vitamin A + vitamin A deficiency* | Intervention NE vs NoNE  MUAC at pre-intervention  K-Score *vitamin A + vitamin A deficiency* at pre-intervention  Hospital  Hospital x MUAC at pre-intervention | - |
| K-Score *total* | Intervention NE vs NoNE  MUAC at pre-intervention  K-Score *total* at pre-intervention  Hospital | - |
| P-Score *iron* | Intervention NE vs NoNE  MUAC at pre-intervention  P-Score *iron* at pre-intervention  Hospital | - |
| DDS with NE vs NoNE | Intervention NE vs NoNE  MUAC at pre-intervention  DDS at pre-intervention  Hospital  Hospital x MUAC at pre-intervention | Hospital x Intervention NE vs NoNE |
| MDD-W with NE vs NoNE | Intervention NE vs NoNE  MUAC at pre-intervention  MDD-W at pre-intervention  Hospital  Hospital x MUAC at pre-intervention | - |
| DDS with six intervention and control groups | Intervention group  DDS at pre-intervention  Hospital | - |
| MDD-W with six intervention and control groups | Intervention group  MDD-W at pre-intervention  Hospital | Hospital x Intervention group |
| Hemoglobin with six intervention and control groups | Intervention group  Hb at pre-intervention  Hospital | - |
| Hemoglobin in anemic mothers with six intervention and control groups | Intervention group  Hb at pre-intervention  Hospital | Hospital x Intervention NE vs NoNE |
| Modification of the own diet | Intervention NE vs NoNE  MUAC at pre-intervention  Hospital | - |
| Modification of breastfeeding behavior | Intervention NE vs NoNE  MUAC at pre-intervention | - |
| Modification of complementary feeding | Intervention NE vs NoNE  MUAC at pre-intervention | - |

^a^ All models include the fixed intercept.

DDS: Dietary diversity score; Hb: Hemoglobin; K-Score: Knowledge score; MDD-W: Minimum Dietary Diversity for Women; MUAC: mid-upper arm circumference; NE: Nutrition education; NoNE: no nutrition education; P-Score: Practice score.

**Table S4.** Nutritional knowledge and practices of mothers in nutrition education group and no nutrition education group at pre- and post-intervention

| **Variables^a^** | **Nutrition education**  ***n*=103** | | **No nutrition education**  ***n*=313** | | **Total^b^**  ***N*=416** | |
| --- | --- | --- | --- | --- | --- | --- |
|  | *n* | % | *n* | % | *n* | % |
| *Knowledge about three food group model* | | |  |  |  |  |
| Knows at least one of the 3 types of food  Pre-intervention  Post-intervention | *n*=97  41  70 | 42.3  72.7 | *n*=308  122  183 | 39.6  59.4 | *n*=405  163  253 | 40.2  62.5 |
| Knows an energy-giving food  Pre-intervention  Post-intervention | *n*=99  38  64 | 38.4  64.6 | *n*=307  119  118 | 38.8  38.4 | *n*=406  157  182 | 38.7  44.8 |
| Knows a constructive food  Pre-intervention  Post-intervention | *n*=100  46  49 | 46.0  49.0 | *n*=308  91  81 | 29.5  26.3 | *n*=408  137  130 | 33.6  31.9 |
| Knows a protective food  Pre-intervention  Post-intervention | *n*=98  35  46 | 35.7  46.9 | *n*=306  82  66 | 26.8  21.6 | *n*=404  117  112 | 29.0  27.7 |
| *Knowledge about malnutrition* |  |  |  |  |  |  |
| Can define malnutrition  Pre-intervention  Post-intervention | *n*=97  72  82 | 74.2  84.5 | *n*=310  218  229 | 70.3  73.9 | *n*=407  290  311 | 71.3  76.4 |
| Knows a consequence/symptom of malnutrition  Pre-intervention  Post-intervention | *n*=101  88  90 | 87.1  89.1 | *n*=312  266  248 | 85.3  79.5 | *n*=413  354  338 | 85.7  81.8 |
| Knows a cause of malnutrition  Pre-intervention  Post-intervention | *n*=100  82  82 | 82.0  82.0 | *n*=312  243  219 | 77.9  70.2 | *n*=412  325  301 | 78.9  73.1 |
| Knows a measure to prevent malnutrition  Pre-intervention  Post-intervention | *n*=102  76  67 | 74.5  65.7 | *n*=309  211  157 | 68.3  50.8 | *n*=411  287  224 | 69.8  54.5 |
| *Knowledge about iron, iron deficiency, anemia* | | | |  |  |  |
| Knows a sign/symptom of anemia^c^  Pre-intervention  Post-intervention | *n*=101  25  53 | 24.8  52.5 | *n*=309  88  105 | 28.5  34.0 | *n*=410  113  158 | 27.6  38.5 |
| Knows a consequence of iron-deficient nutrition in children  Pre-intervention  Post-intervention | *n*=100  29  50 | 29.0  50.0 | *n*=309  68  60 | 22.0  19.4 | *n*=409  97  110 | 23.7  26.9 |
| Knows a cause of anemia  Pre-intervention  Post-intervention | *n*=99  34  52 | 34.3  52.5 | *n*=307  99  110 | 32.2  35.8 | *n*=406  133  162 | 32.8  39.9 |
| Knows a measure to prevent anemia  Pre-intervention  Post-intervention | *n*=102  36  51 | 35.3  50.0 | *n*=309  95  92 | 30.7  29.8 | *n*=411  131  143 | 31.9  34.8 |
| Knows an iron-rich food  Pre-intervention  Post-intervention | *n*=100  35  55 | 35.0  55.0 | *n*=306  95  102 | 31.0  33.3 | *n*=406  130  157 | 32.0  38.7 |
| Knows a food/method increasing iron bioavailability  Pre-intervention  Post-intervention | *n*=99  7  29 | 7.1  29.3 | *n*=299  19  29 | 6.4  9.7 | *n*=398  26  58 | 6.5  14.6 |
| Knows a drink decreasing iron bioavailability  Pre-intervention  Post-intervention | *n*=97  5  28 | 5.2  28.9 | *n*=298  11  13 | 3.7  4.4 | *n*=395  16  41 | 4.1  10.4 |
| *Knowledge about vitamin A, vitamin A deficiency* | | | |  |  |  |
| Knows a sign/symptom of VAD^d^  Pre-intervention  Post-intervention | *n*=99  22  51 | 22.2  51.5 | *n*=306  73  100 | 23.9  32.7 | *n*=405  95  151 | 23.5  37.3 |
| Knows a cause of VAD  Pre-intervention  Post-intervention | *n*=101  33  4 | 32.7  53.5 | *n*=307  124  121 | 36.2  39.4 | *n*=408  157  175 | 35.3  42.9 |
| Knows a measure to prevent of VAD  Pre-intervention  Post-intervention | *n*=101  31  54 | 30.7  53.5 | *n*=308  89  85 | 28.9  27.6 | *n*=409  120  139 | 29.3  34.0 |
| Knows a vitamin A-rich food  Pre-intervention  Post-intervention | *n*=98  36  47 | 36.7  48.0 | *n*=308  105  98 | 34.1  31.8 | *n*=406  141  145 | 34.7  35.7 |
| *Practices regarding iron* |  |  |  |  |  |  |
| Eats fruits with a meal  Pre-intervention  Post-intervention | *n*=101  74  86 | 73.3  85.1 | *n*=310  248  269 | 80.0  86.8 | *n*=411  322  355 | 78.3  86.4 |
| Does not drink coffee/tea with a meal  Pre-intervention  Post-intervention | *n*=98  25  16 | 25.5  16.3 | *n*=310  99  67 | 31.9  21.6 | *n*=408  124  83 | 30.4  20.3 |
| Practices fermentation  Pre-intervention  Post-intervention | *n*=95  1  7 | 1.1  7.4 | *n*=296  3  4 | 1.0  1.4 | *n*=391  4  11 | 1.0  2.8 |
| Practices roasting of flour  Pre-intervention  Post-intervention | *n*=102  2  17 | 2.0  16.7 | *n*=311  11  15 | 2.6  4.8 | *n*=413  10  32 | 2.4  7.7 |
| Eats animal foods at least once per week  Pre-intervention  Post-intervention | *n*=95  77  86 | 81.1  90.5 | *n*=324  227  247 | 74.9  81.5 | *n*=398  304  333 | 76.4  83.7 |
| Eats green leafy vegetables at least 3x per week  Pre-intervention  Post-intervention | *n*=101  78  73 | 77.2  72.3 | *n*=311  223  188 | 71.7  60.5 | *n*=412  301  261 | 73.1  63.3 |

^a^ Categorical variables are expressed as % (n).

^b^ Lack of corresponding sum of frequencies with total sample size is due to missing data; only cases who answered question at both assessments were considered; total frequencies per variable are given.

^c^ Not knowing anemia / VAD considered as not knowing a symptom.

VAD: vitamin A deficiency.

**Table S5.** Knowledge and practice scores of mothers in nutrition education group and no nutrition education group at pre- and post-intervention

| **Variables ^a^** | **Nutrition education**  ***n*=103** | **No nutrition education**  ***n*=313** | **Total ^b^**  ***N*=416** |
| --- | --- | --- | --- |
| *Knowledge scores* |  |  |  |
| K-Score *three food group model*  Pre-intervention  Post-intervention | *n*=99  0.40 ± 0.38  0.25 (0.00, 0.75)  0.57 ± 0.39  0.50 (0.25, 1.00) | *n*=310  0.34 ± 0.34  0.25 (0.00, 0.50)  0.36 ± 0.32  0.25 (0.00, 0.50) | *n*=409  0.35 ± 0.35  0.25 (0.00, 0.75)  0.41 ± 0.34  0.25 (0.25, 0.75) |
| K-Score *malnutrition*  Pre-intervention  Post-intervention | *n*=101  0.79 ± 0.33  1.00 (0.75, 1.00)  0.80 ± 0.28  1.00 (0.75, 1.00) | *n*=312  0.75 ± 0.33  1.00 (0.50, 1.00)  0.69 ± 0.34  0.75 (0.50, 1.00) | *n*=413  0.76 ± 0.33  1.00 (0.50, 1.00)  0.71 ± 0.33  0.75 (0.50, 1.00) |
| K-Score *iron + anemia*  Pre-intervention  Post-intervention | *n*=95  0.25 ± 0.32  0.00 (0.00, 0.50)  0.47 ± 0.37  0.50 (0.00, 0.83) | *n*=290  0.23 ± 0.31  0.00 (0.00, 0.45)  0.24 ± 0.28  0.17 (0.00, 0.43) | *n*=385  0.24 ± 0.31  0.00 (0.00, 0.50)  0.30 ± 0.32  0.17 (0.00, 0.57) |
| K-Score *vitamin A + VAD*  Pre-intervention  Post-intervention | *n*=99  0.33 ± 0.40  0.00 (0.00, 0.67)  0.53 ± 0.42  0.67 (0.00, 1.00) | *n*=305  0.33 ± 0.40  0.00 (0.00, 0.75)  0.35 ± 0.37  0.25 (0.00, 0.75) | *n*=404  0.33 ± 0.40  0.00 (0.00, 0.75)  0.39 ± 0.39  0.33 (0.00, 0.75) |
| K-Score *total*  Pre-intervention  Post-intervention | *n*=100  0.44 ± 0.24  0.39 (0.28, 0.56)  0.59 ±0.28  0.57 (0.36, 0.89) | *n*=310  0.40 ± 0.24  0.39 (0.24, 0.56)  0.40 ± 0.23  0.39 (0.24, 0.56) | *n*=410  0.41 ± 0.24  0.39 (0.24, 0.56)  0.44 ±0.25  0.41 (0.24, 0.63) |
| *Practice scores* |  |  |  |
| P-Score *iron*  Pre-intervention  Post-intervention | *n*=95  0.42 ± 0.15  0.50 (0.33, 0.50)  0.48 ± 0.15  0.50 (0.33, 0.50) | *n*=303  0.43 ± 0.14  0.50 (0.33, 0.50)  0.43 ± 0.14  0.50 (0.33, 0.50) | *n*=398  0.43 ± 0.14  0.50 (0.33, 0.50)  0.44 ±0.14  0.50 (0.33, 0.50) |

^a^ Metric variables are expressed as mean ± SD and median (IQR) and categorical variables are expressed as % (n).
^b^ Lack of corresponding sum of frequencies with total sample size is due to missing data; total frequencies per variable are given.

VAD: vitamin A deficiency.

**Table S6.** LMMs and GLMMs for the change of K- and P-Scores, DDS, and Hb of mothers from pre- to post-intervention, and MDD-W and dietary modifications at post-intervention

|  | ***F*** | ***P*-value** |
| --- | --- | --- |
| *K-Score three food group model (n=408)^a^* |  |  |
| Corrected Model | 44.990 | 0.000* |
| Intervention NE vs NoNE | 23.584 | 0.000* |
| MUAC at pre-intervention | 10.278 | 0.001* |
| K-Score *three food group model* at pre-intervention | 281.573 | 0.000* |
| Hospital | 4.809 | 0.009* |
| Hospital x MUAC at pre-intervention | 4.439 | 0.012* |
| *K-Score malnutrition (n=412)^a^* |  |  |
| Corrected Model | 50.751 | 0.000* |
| Intervention NE vs NoNE | 5.674 | 0.018* |
| MUAC at pre-intervention | 2.077 | 0.150 |
| K-Score *malnutrition* at pre-intervention | 248.484 | 0.000* |
| Hospital | 1.878 | 0.154 |
| *K-Score iron (n=384)^a^* |  |  |
| Corrected Model | 44.117 | 0.000* |
| Intervention NE vs NoNE | 15.172 | 0.106 |
| MUAC at pre-intervention | 6.999 | 0.008* |
| K-Score *iron + anemia* at pre-intervention | 190.462 | 0.000* |
| Hospital | 0.418 | 0.659 |
| *K-Score vitamin A + vitamin A deficiency (n=403)^a^* |  |  |
| Corrected Model | 31.972 | 0.000* |
| Intervention NE vs NoNE | 20.889 | 0.000* |
| MUAC at pre-intervention | 0.239 | 0.625 |
| K-Score *vitamin A + VAD* at pre-intervention | 199.460 | 0.000* |
| Hospital | 1.814 | 0.164 |
| Hospital x MUAC at pre-intervention | 3.861 | 0.022* |
| *K-Score total (n=409)^a^* |  |  |
| Corrected Model | 46.228 | 0.000* |
| Intervention NE vs NoNE | 36.380 | 0.000* |
| MUAC at pre-intervention | 3.547 | 0.060 |
| K-Score *total* at pre-intervention | 193.779 | 0.000* |
| Hospital | 1.917 | 0.148 |
| *P-Score iron (n=397)^a^* |  |  |
| Corrected Model | 65.075 | 0.000* |
| Intervention NE vs NoNE | 9.391 | 0.002* |
| MUAC at pre-intervention | 0.369 | 0.544 |
| P-Score *iron* at pre-intervention | 303.950 | 0.000* |
| Hospital | 4.936 | 0.008* |
| *DDS with NE vs NoNE (n=369)^a^* |  |  |
| Corrected Model | 51.383 | 0.000* |
| Intervention NE vs NoNE | 0.004 | 0.955 |
| MUAC at pre-intervention | 0.004 | 0.950 |
| DDS at pre-intervention | 313.744 | 0.000* |
| Hospital | 9.577 | 0.000* |
| Hospital x MUAC at pre-intervention | 3.167 | 0.043* |
| *MDD-W with NE vs NoNE (n=369)^a^* |  |  |
| Corrected Model | 6.483 | 0.000* |
| Intervention NE vs NoNE | 2.161 | 0.143 |
| MUAC at pre-intervention | 0.004 | 0.949 |
| MDD-W at pre-intervention | 2.128 | 0.146 |
| Hospital | 16.328 | 0.000* |
| Hospital x MUAC at pre-intervention | 6.018 | 0.003* |
| *DDS with six intervention and control groups (n=370)^a^* |  |  |
| Corrected Model | 44.715 | 0.000* |
| Intervention groups | 0.797 | 0.552 |
| DDS at pre-intervention | 333.205 | 0.000* |
| Hospital | 13.386 | 0.000* |
| *MDD-W with six intervention and control groups (n=370)^a^* | |  |
| Corrected Model | 4.210 | 0.040* |
| Intervention groups | 0.699 | 0.646 |
| MDD-W at pre-intervention | 1.167 | 0.281 |
| Hospital | 15.289 | 0.002* |
| *Modification of the own diet (n=412)^a^* |  |  |
| Corrected Model | 2.088 | 0.083 |
| Intervention NE vs NoNE | 1.244 | 0.266 |
| MUAC at pre-intervention | 0.770 | 0.381 |
| Hospital | 2.528 | 0.082 |
| *Modification of breastfeeding behavior (n=408)^a^* |  |  |
| Corrected Model | 6.557 | 0.002* |
| Intervention NE vs NoNE | 0.431 | 0.512 |
| MUAC at pre-intervention | 12.606 | 0.000* |
| *Modification of complementary feeding (n=415)^a^* |  |  |
| Corrected Model | 3.761 | 0.025* |
| Intervention NE vs NoNE | 4.758 | 0.030* |
| MUAC at pre-intervention | 1.257 | 0.263 |
| *Hemoglobin with six intervention and control groups (n=399)* | |  |
| Corrected Model | 15.508 | 0.000* |
| Intervention groups | 0.622 | 0.683 |
| Hb at pre-intervention | 111.241 | 0.000* |
| Hospital | 4.210 | 0.016* |
| *Hemoglobin in anemic mothers with six intervention and control groups (n=111)* | | |
| Corrected Model | 3.912 | 0.031* |
| Intervention groups | 0.233 | 0.934 |
| Hb at pre-intervention | 26.111 | 0.000* |
| Hospital | 2.948 | 0.108 |

^a^ Lack of corresponding sum of frequencies with total sample size is due to missing data; total frequencies per variable are given.

* Significant effect.

DDS: Dietary diversity score; Hb: Hemoglobin; K-Score: Knowledge score; MDD-W: Minimum Dietary Diversity for Women; MUAC: Mid-upper arm circumference; NE: Nutrition education; NoNE: No nutrition education; P-Score: Practice score; VAD: Vitamin A deficiency.

**Table S7.** Fixed coefficients of the LMMs for the change of K- and P-Scores from pre- to post-intervention

|  | **Coefficient** | **SE** | ***P*-value** | **95% CI** | |
| --- | --- | --- | --- | --- | --- |
|  |  |  |  | **Lower** | **Upper** |
| *K-Score three food group model (n=408)^a^* |  |  |  |  |  |
| Intercept | -0.004 | 0.032 | 0.889 | -0.067 | 0.058 |
| NE-group^b^ | 0.181 | 0.037 | 0.000* | 0.108 | 0.254 |
| MUAC at pre-intervention | 0.017 | 0.013 | 0.198 | -0.009 | 0.042 |
| K-Score *three food group model* at pre-intervention | -0.804 | 0.048 | 0.000* | -0.899 | -0.710 |
| Hospital 1 (Nyantende)^c^ | 0.169 | 0.057 | 0.003 | 0.057 | 0.282 |
| Hospital 2 (Ciriri)^c^ | 0.013 | 0.037 | 0.718 | -0.059 | 0.086 |
| MUAC at pre-intervention x Hospital 1^c^ | 0.034 | 0.020 | 0.080 | -0.004 | 0.073 |
| MUAC at pre-intervention x Hospital 2^c^ | -0.016 | 0.015 | 0.311 | -0.046 | 0.015 |
| *K-Score malnutrition (n=412)^a^* |  |  |  |  |  |
| Intercept | -0.041 | 0.030 | 0.182 | -0.100 | 0.019 |
| NE-group^b^ | 0.087 | 0.036 | 0.018* | 0.015 | 0.158 |
| MUAC at pre-intervention | 0.009 | 0.006 | 0.150 | -0.003 | 0.021 |
| K-Score *malnutrition* at pre-intervention | -0.739 | 0.047 | 0.000* | -0.831 | -0.647 |
| Hospital 1 (Nyantende)^c^ | 0.019 | 0.054 | 0.728 | -0.087 | 0.124 |
| Hospital 2 (Ciriri)^c^ | -0.056 | 0.035 | 0.115 | -0.126 | 0.014 |
| *K-Score iron + anemia (n=384)^a^* |  |  |  |  |  |
| Intercept | -0.012 | 0.050 | 0.853 | -0.544 | 0.521 |
| NE-group^b^ | 0.209 | 0.054 | 0.106 | -0.160 | 0.577 |
| MUAC at pre-intervention | 0.016 | 0.006 | 0.008* | 0.004 | 0.027 |
| K-Score *iron + anemia* at pre-intervention | -0.677 | 0.049 | 0.000* | -0.774 | -0.581 |
| Hospital 1 (Nyantende)^c^ | 0.066 | 0.073 | 0.451 | -0.225 | 0.358 |
| Hospital 2 (Ciriri)^c^ | 0.025 | 0.060 | 0.749 | -0.687 | 0.737 |
| *K-Score vitamin A + vitamin A deficiency (n=403)^a^* | |  |  |  |  |
| Intercept | 0.083 | 0.036 | 0.021* | 0.013 | 0.153 |
| NE-group^b^ | 0.189 | 0.041 | 0.000* | 0.108 | 0.270 |
| MUAC at pre-intervention | 0.028 | 0.014 | 0.056 | -0.001 | 0.056 |
| K-Score *vitamin A + VAD* at pre-intervention | -0.620 | 0.044 | 0.000* | -0.706 | -0.533 |
| Hospital 1 (Nyantende)^c^ | -0.057 | 0.062 | 0.360 | -0.179 | 0.065 |
| Hospital 2 (Ciriri)^c^ | -0.078 | 0.041 | 0.058 | -0.159 | 0.003 |
| MUAC at pre-intervention x Hospital 1^c^ | -0.025 | 0.022 | 0.256 | -0.067 | 0.018 |
| MUAC at pre-intervention x Hospital 2^c^ | -0.047 | 0.017 | 0.007* | -0.080 | -0.013 |
| *K-Score total (n=409)^a^* |  |  |  |  |  |
| Intercept | 0.001 | 0.022 | 0.975 | -0.043 | 0.044 |
| NE-group^b^ | 0.161 | 0.027 | 0.000* | 0.108 | 0.213 |
| MUAC at pre-intervention | 0.009 | 0.005 | 0.060 | 0.000 | 0.018 |
| K-Score *total* at pre-intervention | -0.687 | 0.049 | 0.000* | -0.784 | -0.590 |
| Hospital 1 (Nyantende)^c^ | 0.053 | 0.040 | 0.181 | -0.025 | 0.132 |
| Hospital 2 (Ciriri)^c^ | -0.020 | 0.026 | 0.440 | -0.071 | 0.031 |
| *P-Score iron (n=397)^a^* |  |  |  |  |  |
| Intercept | 0.029 | 0.014 | 0.031* | 0.003 | 0.056 |
| NE-group^b^ | 0.051 | 0.017 | 0.002* | 0.018 | 0.083 |
| MUAC at pre-intervention | 0.002 | 0.003 | 0.544 | -0.004 | 0.007 |
| P-Score *iron* at pre-intervention | -0.855 | 0.049 | 0.000* | -0.951 | -0.758 |
| Hospital 1 (Nyantende)^c^ | -0.025 | 0.025 | 0.304 | -0.073 | 0.023 |
| Hospital 2 (Ciriri)^c^ | -0.050 | 0.016 | 0.002 | -0.081 | -0.019 |

^a^ Lack of corresponding sum of frequencies with total sample size is due to missing data; total frequencies per variable are given.

^b^ Reference category was NoNE-group.

^c^ Reference category was Hospital 3 (Nyangezi).

* Significant effect.

K-Score: Knowledge score; NE: Nutrition education; NoNE: No nutrition education; P-Score: Practice score; VAD: Vitamin A deficiency.

**Table S8.** Fixed coefficients of the LMMs and GLMMs for the change of DDS from pre- to post-intervention and MDD-W at post-intervention

|  | **Coefficient** | **SE** | ***P*-value** | **95% CI** | | **OR** | **95% CI** | |
| --- | --- | --- | --- | --- | --- | --- | --- | --- |
|  |  |  |  | **Lower** | **Upper** |  | **Lower** | **Upper** |
| *DDS with NE vs NoNE (n=369)^a^* | |  |  |  |  |  |  |  |
| Intercept | 0.109 | 0.149 | 0.588 | -1.383 | 1.601 |  |  |  |
| NE-group^b^ | 0.011 | 0.162 | 0.955 | -1.059 | 1.081 |  |  |  |
| MUAC at pre-intervention | 0.047 | 0.043 | 0.278 | -0.038 | 0.131 |  |  |  |
| DDS at pre-intervention | -0.857 | 0.048 | 0.000* | -0.952 | -0.762 |  |  |  |
| Hospital 1 (Nyantende)^c^ | 0.637 | 0.222 | 0.083 | -0.180 | 1.455 |  |  |  |
| Hospital 2 (Ciriri)^c^ | -0.287 | 0.179 | 0.355 | -2.578 | 2.004 |  |  |  |
| MUAC at  pre-intervention x Hospital 1^c^ | -0.137 | 0.066 | 0.039* | -0.267 | -0.007 |  |  |  |
| MUAC at  pre-intervention x Hospital 2^c^ | 0.001 | 0.051 | 0.979 | -0.098 | 0.101 |  |  |  |
| *MDD-W with NE vs NoNE (n=369)^a^* | |  |  |  |  |  |  |  |
| Intercept | -2.745 | 0.445 | 0.000* | -3.621 | -1.869 | 0.064 | 0.027 | 0.154 |
| NE-group^b^ | -0.630 | 0.428 | 0.143 | -1.474 | 0.214 | 0.533 | 0.229 | 1.239 |
| MUAC at pre-intervention | 0.164 | 0.200 | 0.413 | -0.229 | 0.557 | 1.178 | 0.795 | 1.745 |
| MDD-W at pre-intervention^d^ | 0.618 | 0.423 | 0.146 | -0.216 | 1.451 | 1.854 | 0.806 | 4.269 |
| Hospital 1 (Nyantende)^c^ | 2.678 | 0.552 | 0.000* | 1.592 | 3.764 | 14.554 | 4.912 | 43.124 |
| Hospital 2 (Ciriri)^c^ | 0.475 | 0.495 | 0.338 | -0.500 | 1.451 | 1.609 | 0.606 | 4.268 |
| MUAC at  pre-intervention x Hospital 1^c^ | -0.531 | 0.245 | 0.031* | -1.013 | -0.049 | 0.588 | 0.363 | 0.953 |
| MUAC at  pre-intervention x Hospital 2^c^ | 0.023 | 0.215 | 0.915 | -0.400 | 0.446 | 1.023 | 0.670 | 1.562 |
| *DDS with six intervention and control groups (n=370)^a^* | | | |  |  |  |  |  |
| Intercept | -0.029 | 0.122 | 0.811 | -0.270 | 0.211 |  |  |  |
| LM-NE^e^ | 0.017 | 0.185 | 0.926 | -0.346 | 0.380 |  |  |  |
| LM-S^e^ | 0.279 | 0.180 | 0.123 | -0.076 | 0.633 |  |  |  |
| LM-C^e^ | -0.027 | 0.187 | 0.886 | -0.393 | 0.340 |  |  |  |
| HM-NE^e^ | 0.188 | 0.157 | 0.234 | -0.122 | 0.497 |  |  |  |
| HM-C^e^ | 0.149 | 0.160 | 0.355 | -0.167 | 0.464 |  |  |  |
| DDS at pre-intervention | -0.867 | 0.048 | 0.000* | -0.960 | -0.773 |  |  |  |
| Hospital 1 (Nyantende)^c^ | 0.684 | 0.180 | 0.000* | 0.330 | 1.037 |  |  |  |
| Hospital 2 (Ciriri)^c^ | -0.190 | 0.120 | 0.114 | -0.427 | -0.046 |  |  |  |
| *MDD-W with six intervention and control groups (n=370)^a^* | | | |  |  |  |  |  |
| Intercept | -3.102 | 0.553 | 0.000* | -4.375 | -1.829 | 0.045 | 0.013 | 0.161 |
| LM-NE^e^ | 0.281 | 0.648 | 0.679 | -1.286 | 1.847 | 1.324 | 0.276 | 6.340 |
| LM-S^e^ | 0.320 | 0.652 | 0.640 | -1.268 | 1.909 | 1.378 | 0.281 | 6.749 |
| LM-C^e^ | 0.183 | 0.685 | 0.796 | -1.397 | 1.763 | 1.201 | 0.247 | 5.829 |
| HM-NE^e^ | -0.666 | 0.670 | 0.658 | -2.305 | 0.973 | 0.514 | 0.100 | 2.645 |
| HM-C^e^ | 0.563 | 0.562 | 0.394 | -1.287 | 2.413 | 1.756 | 0.276 | 11.171 |
| MDD-W at  pre-intervention^d^ | 0.457 | 0.423 | 0.281 | -0.375 | 1.288 | 1.579 | 0.687 | 3.627 |
| Hospital 1 (Nyantende)^c^ | 2.819 | 0.569 | 0.000* | 1.609 | 4.028 | 16.753 | 4.997 | 56.168 |
| Hospital 2 (Ciriri)^c^ | 0.805 | 0.530 | 0.159 | -0.373 | 1.984 | 2.238 | 0.689 | 7.272 |

^a^ Lack of corresponding sum of frequencies with total sample size is due to missing data; total frequencies per variable are given.

^b^ Reference category was NoNE-group.

^c^ Reference category was Hospital 3 (Nyangezi).

^d^ Reference category was MDD-W not reached (< 5 food groups).

^e^ Reference category was NM-C-group.

* Significant effect.

DDS: Dietary diversity score; HM-C: High MUAC–control; HM-NE: High MUAC–nutrition education; LM-C: Low MUAC–control; LM-NE: Low MUAC–nutrition education; LM-S: Low MUAC–supplement; MDD-W: Minimum Dietary Diversity for Women; MUAC: mid-upper arm circumference; NE: Nutrition education; NM-C: Normal MUAC–control; NoNE: No nutrition education

**Table S9.** Estimated mean differences in the change of DDS from pre- to post-intervention between the six intervention and control groups according to the LMMs

|  | **Mean difference** | **SE** | **95% CI** | | ***P*-value^b^** |
| --- | --- | --- | --- | --- | --- |
|  |  |  | **Lower** | **Upper** |  |
| *DDS with six intervention and control groups (n=370)^a^* | | | |  |  |
| LM-NE - LM-S | -0.262 | 0.226 | -0.706 | 0.183 | 0.248 |
| LM-NE - LM-C | 0.044 | 0.230 | -0.409 | 0.496 | 0.849 |
| LM-NE - NM-C | 0.017 | 0.185 | -0.346 | 0.380 | 0.926 |
| LM-NE - HM-NE | -0.171 | 0.211 | -0.585 | 0.244 | 0.419 |
| LM-NE - HM-C | -0.132 | 0.213 | -0.551 | 0.288 | 0.538 |
| LM-S - LM-NE | 0.262 | 0.226 | -0.183 | 0.706 | 0.248 |
| LM-S - LM-C | 0.305 | 0.227 | -0.142 | 0.752 | 0.180 |
| LM-S - NM-C | 0.279 | 0.180 | -0.076 | 0.633 | 0.123 |
| LM-S - HM-NE | 0.091 | 0.208 | -0.318 | 0.500 | 0.662 |
| LM-S - HM-C | 0.130 | 0.211 | -0.284 | 0.544 | 0.538 |
| LM-C - LM-NE | -0.044 | 0.230 | -0.496 | 0.409 | 0.849 |
| LM-C - LM-S | -0.305 | 0.227 | -0.752 | 0.142 | 0.180 |
| LM-C - NM-C | -0.027 | 0.186 | -0.393 | 0.340 | 0.886 |
| LM-C - HM-NE | -0.214 | 0.213 | -0.633 | 0.204 | 0.315 |
| LM-C - HM-C | -0.175 | 0.215 | -0.599 | 0.248 | 0.416 |
| NM-C - LM-NE | -0.017 | 0.185 | -0.380 | 0.346 | 0.926 |
| NM-C - LM-S | -0.279 | 0.180 | -0.633 | 0.076 | 0.123 |
| NM-C - LM-C | 0.027 | 0.186 | -0.340 | 0.393 | 0.886 |
| NM-C - HM-NE | -0.188 | 0.157 | -0.497 | 0.122 | 0.234 |
| NM-C - HM-C | -0.149 | 0.160 | -0.464 | 0.167 | 0.355 |
| HM-NE - LM-NE | 0.171 | 0.211 | -0.244 | 0.585 | 0.419 |
| HM-NE - LM-S | -0.091 | 0.208 | -0.500 | 0.318 | 0.662 |
| HM-NE - LM-C | 0.214 | 0.213 | -0.204 | 0.633 | 0.315 |
| HM-NE - NM-C | 0.188 | 0.157 | -0.122 | 0.497 | 0.234 |
| HM-NE - HM-C | 0.039 | 0.189 | -0.333 | 0.411 | 0.837 |
| HM-C - LM-NE | 0.132 | 0.213 | -0.288 | 0.551 | 0.538 |
| HM-C - LM-S | -0.130 | 0.211 | -0.544 | 0.284 | 0.538 |
| HM-C - LM-C | 0.175 | 0.215 | -0.248 | 0.599 | 0.416 |
| HM-C - NM-C | 0.149 | 0.160 | -0.167 | 0.464 | 0.355 |
| HM-C - HM-NE | -0.039 | 0.189 | -0.411 | 0.333 | 0.837 |

^a^ Lack of corresponding sum of frequencies with total sample size is due to missing data; total frequency is given.

^b^ Adjusted significance.

DDS: Dietary diversity score; HM-C: High MUAC–control; HM-NE: High MUAC–nutrition education; LM-C: Low MUAC–control; LM-NE: Low MUAC–nutrition education; LM-S: Low MUAC–supplement; NM-C: Normal MUAC–control.

**Table S10.** Fixed effects of the GLMMs for dietary modifications at post-intervention

|  | **Coefficient** | **SE** | ***P*-value** | **95% CI** | | **OR** | **95% CI** | |
| --- | --- | --- | --- | --- | --- | --- | --- | --- |
|  |  |  |  | **Lower** | **Upper** |  | **Lower** | **Upper** |
| *Modification of the own diet (n=412)^a, b^* | | |  |  |  |  |  |  |
| Intercept | -3.097 | 0.474 | 0.000* | -4.031 | -2.163 | 0.045 | 0.018 | 0.115 |
| NE-group^c^ | 0.428 | 0.383 | 0.266 | -0.328 | 1.183 | 1.534 | 0.721 | 3.263 |
| MUAC at  pre-intervention | 0.058 | 0.066 | 0.381 | -0.072 | 0.189 | 1.060 | 0.930 | 1.208 |
| Hospital 1 (Nyantende)^d^ | 1.183 | 0.634 | 0.063 | -0.065 | 2.431 | 3.266 | 0.937 | 11.375 |
| Hospital 2 (Ciriri)^d^ | 1.088 | 0.502 | 0.031* | 0.099 | 2.077 | 2.968 | 1.104 | 7.980 |
| *Modification of breastfeeding behavior (n=408)^a^* | | | |  |  |  |  |  |
| Intercept | -2.623 | 0.274 | 0.000* | -3.164 | -2.082 | 0.073 | 0.042 | 0.125 |
| NE-group^c^ | -0.359 | 0.546 | 0.512 | -1.436 | 0.719 | 0.699 | 0.238 | 2.052 |
| MUAC at  pre-intervention | 0.308 | 0.087 | 0.000* | 0.137 | 0.479 | 1.360 | 1.147 | 1.614 |
| *Modification of complementary feeding (n=415)^a^* | | | |  |  |  |  |  |
| Intercept | -3.306 | 0.332 | 0.000* | -3.962 | -2.651 | 0.037 | 0.019 | 0.071 |
| NE-group^c^ | 1.075 | 0.493 | 0.030* | 0.103 | 2.047 | 2.930 | 1.109 | 7.742 |
| MUAC at  pre-intervention | 0.092 | 0.082 | 0.263 | -0.070 | 0.254 | 1.097 | 0.932 | 1.290 |

^a^ Lack of corresponding sum of frequencies with total sample size is due to missing data; total frequencies per variable are given.

^b^ One mother reporting “do not know” was excluded from analysis.

^c^ Reference category was NoNE-group.

^c^ Reference category was Hospital 3 (Nyangezi).

* Significant effect.

MUAC: Mid-upper arm circumference; NE: Nutrition education; NoNE: No nutrition education.

**Table S11.** Fixed effects of the LMMs for the change of hemoglobin from pre- to post-intervention

|  | **Coefficient** | **SE** | ***P*-value** | **95% CI** | |  |
| --- | --- | --- | --- | --- | --- | --- |
|  |  |  |  | **Lower** | **Upper** |  |
| *All mothers (n=399)^a^* |  |  |  |  |  | |
| Intercept | -0.173 | 0.144 | 0.231 | -0.456 | 0.110 | |
| LM-NE^b^ | 0.245 | 0.217 | 0.261 | -0.182 | 0.672 | |
| LM-S^b^ | 0.020 | 0.201 | 0.920 | -0.375 | 0.416 | |
| LM-C^b^ | 0.158 | 0.207 | 0.447 | -0.250 | 0.566 | |
| HM-NE^b^ | 0.220 | 0.186 | 0.238 | -0.146 | 0.586 | |
| HM-C^b^ | -0.057 | 0.191 | 0.766 | -0.432 | 0.319 | |
| Hemoglobin at pre-intervention | -0.438 | 0.042 | 0.000* | -0.520 | -0.356 | |
| Hospital 1 (Nyantende)^c^ | 0.591 | 0.212 | 0.006* | 0.174 | 1.008 | |
| Hospital 2 (Ciriri)^c^ | 0.068 | 0.141 | 0.630 | -0.210 | 0.346 | |
| *Mothers with anemia at pre-intervention (n=111)^a^* | |  |  |  |  | |
| Intercept | 0.424 | 0.261 | 0.221 | -0.510 | 1.357 | |
| LM-NE^b^ | 0.313 | 0.473 | 0.525 | -0.758 | 1.384 | |
| LM-S^b^ | 0.306 | 0.442 | 0.514 | -0.772 | 1.385 | |
| LM-C^b^ | 0.221 | 0.420 | 0.616 | -0.786 | 1.228 | |
| HM-NE^b^ | 0.292 | 0.368 | 0.471 | -0.716 | 1.299 | |
| HM-C^b^ | 0.286 | 0.443 | 0.539 | -0.766 | 1.338 | |
| Hemoglobin at pre-intervention | -0.626 | 0.123 | 0.000* | -0.869 | -0.383 | |
| Hospital 1 (Nyantende)^c^ | 1.514 | 0.635 | 0.021* | 0.237 | 2.791 | |
| Hospital 2 (Ciriri)^c^ | 0.278 | 0.273 | 0.355 | -0.425 | 0.982 | |

^a^ Lack of corresponding sum of frequencies with total sample size is due to missing data; total frequencies per variable are given.

^b^ Reference category was NM-C-group.

^c^ Reference category was Hospital 3 (Nyangezi).

* Significant effect.

HM-C: High MUAC–control; HM-NE: High MUAC–nutrition education; LM-C: Low MUAC–control; LM-NE: Low MUAC–nutrition education; LM-S: Low MUAC–supplement; NM-C: Normal MUAC–control.

**Table S12.** Estimated mean differences in the change of hemoglobin from pre- to post-intervention between the six intervention and control groups according to the LMMs

|  | **Mean difference** | **SE** | **95% CI** | | ***P*-value** |  |
| --- | --- | --- | --- | --- | --- | --- |
|  |  |  | **Lower** | **Upper** |  |  |
| *All mothers (n=399)^a^* |  |  |  |  |  |  |
| LM-NE - LM-S | 0.225 | 0.258 | -0.282 | 0.732 | 0.384 |  |
| LM-NE - LM-C | 0.087 | 0.261 | -0.427 | 0.601 | 0.739 |  |
| LM-NE - NM-C | 0.245 | 0.217 | -0.182 | 0.672 | 0.261 |  |
| LM-NE - HM-NE | 0.025 | 0.249 | -0.464 | 0.514 | 0.920 |  |
| LM-NE - HM-C | 0.302 | 0.253 | -0.196 | 0.799 | 0.234 |  |
| LM-S - LM-NE | -0.225 | 0.258 | -0.732 | 0.282 | 0.384 |  |
| LM-S - LM-C | -0.138 | 0.250 | -0.628 | 0.353 | 0.582 |  |
| LM-S - NM-C | 0.020 | 0.201 | -0.375 | 0.416 | 0.920 |  |
| LM-S - HM-NE | -0.200 | 0.236 | -0.663 | 0.263 | 0.397 |  |
| LM-S - HM-C | 0.077 | 0.239 | -0.393 | 0.548 | 0.747 |  |
| LM-C - LM-NE | -0.087 | 0.261 | -0.601 | 0.427 | 0.739 |  |
| LM-C - LM-S | 0.138 | 0.250 | -0.353 | 0.628 | 0.582 |  |
| LM-C - NM-C | 0.158 | 0.207 | -0.250 | 0.566 | 0.447 |  |
| LM-C - HM-NE | -0.062 | 0.241 | -0.535 | 0.411 | 0.796 |  |
| LM-C - HM-C | 0.215 | 0.245 | -0.266 | 0.695 | 0.380 |  |
| NM-C - LM-NE | -0.245 | 0.217 | -0.672 | 0.182 | 0.261 |  |
| NM-C - LM-S | -0.020 | 0.201 | -0.416 | 0.375 | 0.920 |  |
| NM-C - LM-C | -0.158 | 0.207 | -0.566 | 0.250 | 0.447 |  |
| NM-C - HM-NE | -0.220 | 0.186 | -0.586 | 0.146 | 0.238 |  |
| NM-C - HM-C | 0.057 | 0.191 | -0.319 | 0.432 | 0.766 |  |
| HM-NE - LM-NE | -0.025 | 0.249 | -0.514 | 0.464 | 0.920 |  |
| HM-NE - LM-S | 0.200 | 0.236 | -0.263 | 0.663 | 0.397 |  |
| HM-NE - LM-C | 0.062 | 0.241 | -0.411 | 0.535 | 0.796 |  |
| HM-NE - NM-C | 0.220 | 0.186 | -0.146 | 0.586 | 0.238 |  |
| HM-NE - HM-C | 0.227 | 0.228 | -0.171 | 0.724 | 0.224 |  |
| HM-C - LM-NE | -0.302 | 0.253 | -0.799 | 0.196 | 0.234 |  |
| HM-C - LM-S | -0.077 | 0.239 | -0.548 | 0.393 | 0.747 |  |
| HM-C - LM-C | -0.215 | 0.245 | -0.695 | 0.266 | 0.380 |  |
| HM-C - NM-C | -0.057 | 0.191 | -0.432 | 0.319 | 0.766 |  |
| HM-C - HM-NE | -0.277 | 0.228 | -0.724 | 0.171 | 0.224 |  |
| *Mothers with anemia at pre-intervention (n=111)^a^* | | | | |  |  |
| LM-NE - LM-S | | 0.006 | 0.575 | -1.212 | 1.224 | 0.991 |
| LM-NE - LM-C | | 0.092 | 0.561 | -1.091 | 1.274 | 0.872 |
| LM-NE - NM-C | | 0.313 | 0.473 | -0.758 | 1.384 | 0.525 |
| LM-NE - HM-NE | | 0.021 | 0.523 | -1.102 | 1.144 | 0.969 |
| LM-NE - HM-C | | 0.027 | 0.572 | -1.180 | 1.233 | 0.963 |
| LM-S - LM-NE | | -0.006 | 0.575 | -1.224 | 1.212 | 0.991 |
| LM-S - LM-C | | 0.085 | 0.534 | -1.069 | 1.240 | 0.876 |
| LM-S - NM-C | | 0.306 | 0.442 | -0.772 | 1.385 | 0.514 |
| LM-S - HM-NE | | 0.015 | 0.494 | -1.087 | 1.117 | 0.977 |
| LM-S - HM-C | | 0.020 | 0.551 | -1.166 | 1.207 | 0.971 |
| LM-C - LM-NE | | -0.092 | 0.561 | -1.274 | 1.091 | 0.872 |
| LM-C - LM-S | | -0.085 | 0.534 | -1.240 | 1.069 | 0.876 |
| LM-C - NM-C | | 0.221 | 0.420 | -0.786 | 1.228 | 0.616 |
| LM-C - HM-NE | | -0.071 | 0.472 | -1.109 | 0.968 | 0.884 |
| LM-C - HM-C | | -0.065 | 0.534 | -1.210 | 1.080 | 0.905 |
| NM-C - LM-NE | | -0.313 | 0.473 | -1.384 | 0.758 | 0.525 |
| NM-C - LM-S | | -0.306 | 0.442 | -1.385 | 0.772 | 0.514 |
| NM-C - LM-C | | -0.221 | 0.420 | -1.228 | 0.786 | 0.616 |
| NM-C - HM-NE | | -0.292 | 0.368 | -1.299 | 0.716 | 0.471 |
| NM-C - HM-C | | -0.286 | 0.443 | -1.338 | 0.766 | 0.539 |
| HM-NE - LM-NE | | -0.021 | 0.523 | -1.144 | 1.102 | 0.969 |
| HM-NE - LM-S | | -0.015 | 0.494 | -1.117 | 1.087 | 0.977 |
| HM-NE - LM-C | | 0.071 | 0.472 | -0.968 | 1.109 | 0.884 |
| HM-NE - NM-C | | 0.292 | 0.368 | -0.716 | 1.299 | 0.471 |
| HM-NE - HM-C | | 0.006 | 0.495 | -1.085 | 1.096 | 0.991 |
| HM-C - LM-NE | | -0.027 | 0.572 | -1.233 | 1.180 | 0.963 |
| HM-C - LM-S | | -0.020 | 0.551 | -1.207 | 1.166 | 0.971 |
| HM-C - LM-C | | 0.065 | 0.534 | -1.080 | 1.210 | 0.905 |
| HM-C - NM-C | | 0.286 | 0.443 | -0.766 | 1.338 | 0.539 |
| HM-C - HM-NE | | -0.006 | 0.495 | -1.096 | 1.085 | 0.991 |

^a^ Lack of corresponding sum of frequencies with total sample size is due to missing data; total frequency is given.

^b^ Adjusted significance.

DDS: Dietary diversity score; HM-C: High MUAC–control; HM-NE: High MUAC–nutrition education; LM-C: Low MUAC–control; LM-NE: Low MUAC–nutrition education; LM-S: Low MUAC–supplement; NM-C: Normal MUAC–control.

**Table S13.** Random effect covariance of the LMMs for the change of K- and P-Scores, DDS, and Hb of mothers from pre- to post-intervention

|  | **Estimate** | **SE** | ***P*-value** | **Lower** | **Upper** |
| --- | --- | --- | --- | --- | --- |
| *K-Score iron (n=384)^a^* |  |  |  |  |  |
| Hospital x Intervention NE vs NoNE | 0.002 | 0.005 | 0.647 | 0.000 | 0.161 |
| *DDS with NE vs NoNE (n=369)^a^* |  |  |  |  |  |
| Hospital x Intervention NE vs NoNE | 0.015 | 0.043 | 0.719 | 0.000 | 3.593 |
| *MDD-W with six intervention and control groups (n=370)^a^* | |  |  |  |  |
| Hospital x Intervention groups | 0.117 | 0.320 | 0.713 | 0.001 | 24.383 |
| *Hemoglobin in anemic mothers (n=111)^a^* |  |  |  |  |  |
| Hospital x Intervention groups | 0.008 | 0.109 | 0.938 | 0.000 | 794112651 |

^a^ Lack of corresponding sum of frequencies with total sample size is due to missing data; total frequencies per variable are given.

**Table S14.** Dietary diversity of mothers in the six intervention and control groups at pre- and post-intervention

| **Variables ^a^** | **Low MUAC – nutrition education**  ***n*=38** | **Low MUAC – supplement**  ***n*=40** | **Low MUAC – control**  ***n*=37** | **Normal MUAC – control**  ***n*=144** | **High MUAC – nutrition education**  ***n*=57** | **High MUAC – control**  ***n*=54** | **Total ^b^**  ***N*=370** |
| --- | --- | --- | --- | --- | --- | --- | --- |
| *Dietary diversity score* |  |  |  |  |  |  |  |
| DDS pre-intervention  DDS post-intervention | 3.3 ± 1.2  3.0 (3.0, 4.0)  3.4 ± 1.2  3.0 (2.8, 4.0) | 3.1 ± 0.9  3.0 (2.0, 4.0)  3.6 ± 1.0  3.0 (3.0, 4.0) | 3.2 ± 1.0  3.0 (2.5, 4.0)  3.3 ± 0.9  3.0 (3.0, 4.0) | 3.3 ± 1.1  3.0 (3.0, 4.0)  3.2 ± 1.1  3.0 (2.0, 4.0) | 3.6 ± 1.2  4.0 (3.0, 4.0)  3.5 ± 0.8  4.0 (3.0, 4.0) | 3.7 ± 1.2  4.0 (3.0, 5.0)  3.5 ± 1.1  3.0 (3.0, 4.0) | 3.4 ± 1.1  3.0 (3.0, 4.0)  3.4 ± 1.0  3.0 (3.0, 4.0) |
| *Minimum dietary diversity* |  |  |  |  |  |  |  |
| MDD-W pre-intervention  ≥ 5 food groups  < 5 food groups | 15.8 (6)  84.2 (32) | 10.0 (4)  90.0 (36) | 10.8 (4)  89.2 (33) | 11.1 (16)  88.9 (128) | 22.8 (13)  77.2 (44) | 27.8 (15)  72.2 (39) | 15.7 (58)  84.3 (312) |
| MDD-W post-intervention  ≥ 5 food groups  < 5 food groups | 15.8 (6)  84.2 (32) | 15.0 (6)  85.0 (34) | 13.5 (5)  86.5 (32) | 10.4 (15)  89.6 (129) | 8.8 (5)  91.2 (52) | 22.2 (12)  77.8 (42) | 13.2 (49)  86.8 (321) |

^a^ Metric variables are expressed as mean ± SD and median (IQR).
^b^ Lack of corresponding sum of frequencies with total sample size is due to missing data; total frequencies per variable are given.

DDS: dietary diversity score, MDD-W: minimum dietary diversity for women.

**Table S15*.*** Self-reported modifications of own diet and reasons for modification by mothers in nutrition education group and no nutrition education group at post-intervention

| Modification ^a^ | *n* | Reason ^a^ |
| --- | --- | --- |
| *Nutrition education group (n=15)* |  |  |
| Eating less, reduced number of meals | 7 | Limited financial means / no work (n=6); mother leaves the home |
| Changed consumption of animal foods | 2 | Iron content; due to appetite / taste / wish |
| Changed consumption of vegetables | 2 | Iron content (n=2) |
| Fermentation (flour for porridge) | 1 | According to teaching |
| Changed consumption of staples | 1 | Due to appetite / taste / wish |
| Changed consumption of seeds | 1 | Due to appetite / taste / wish |
| Changed consumption of condiments (chili) | 1 | For breast milk (quantity/quality) |
| Missing | 1 | According to availability |
| *No nutrition education group (n=30)* |  |  |
| Eating less, reduced number of meals | 15 | Limited financial means / no work (n=8); mother leaves the home (n=4); according to availability; availability/affordability; due to appetite / taste / wish; stomach pain |
| Changed consumption of vegetables | 4 | For increase of blood (n=2); for breast milk (quantity/quality); for good health; had amoeba |
| Changed consumption of porridge (including type / preparation) | 4 | For breast milk (quantity/quality) |
| Changed consumption of animal foods | 3 | For breast milk (quantity/quality); for increase of blood; since pregnancy |
| Reduced consumption of tea (including hot water + sugar + soy) | 3 | Mother leaves the home (n=2); availability/affordability |
| Eating more | 2 | According to availability; due to appetite / taste / wish |
| Changed consumption of pulses (milk of soy) | 1 | For breast milk (quantity/quality) |

^a^ Multiple response question.

**Table S16.** Self-reported modifications of breastfeeding behavior and reasons for modification by mothers in nutrition education group and no nutrition education group at post-intervention

| Modification ^a^ | *n* | Reason ^a^ |
| --- | --- | --- |
| *Nutrition education group (n=9)* |  |  |
| Decrease | 6 | Child not always with mother (n=4); food becomes rare; start of complementary food; child grown up |
| Increase | 1 | Due to hunger/crying |
| Introduction of complementary foods | 1 | Due to hunger/crying |
| Missing | 1 | Start of complementary food |
| *No nutrition education group (n=23)* |  |  |
| Decrease | 17 | Child not always with mother (n=14); start of complementary food (n=2); child grown up |
| Increase | 3 | Due to hunger/crying; for strength and growth; will of the child |
| Introduction of complementary foods | 1 | Child not always with mother |
| Stopped breastfeeding | 1 | Will of the child |
| Missing | 2 | Child not always with mother (n=2) |

^a^ Multiple response question.

**Table S17.** Self-reported modifications of complementary feeding and reasons for modification by mothers in nutrition education group and no nutrition education group at post-intervention

| Modification ^a^ | *n* | Reason ^a^ |
| --- | --- | --- |
| *Nutrition education group (n=11)* |  |  |
| Changed porridge (addition of vegetables and/or fruits, plantain banana, dense porridge) | 4 | For satisfaction; for strength/energy; learned in health center/study; iron content |
| Roasting of flour (for porridge) | 3 | Learned in health center/study (n=3) |
| Porridge and palm oil | 1 | Vitamin A content |
| Porridge of maize+sorghum+soy + insect flour | 1 | For antibodies |
| Missing | 2 | Learned in health center/study; complementary food |
| *No nutrition education group (n=11)* |  |  |
| Start, increase in giving porridge | 3 | Child not always with mother; child is 6 months old; without specific reason |
| Changed porridge (addition of powdered milk + sugared biscuits) | 1 | For health |
| Roasting of flour (for porridge) | 1 | Learned at health center |
| Porridge of plantain banana | 1 | Like own diet |
| Porridge of sorghum + soy | 1 | Child cried |
| Porridge of soy + plantain banana | 1 | Child not always with mother |
| Soft ugali of manioc | 1 | For satisfaction |
| Flour of small fish | 1 | Learned that good for health |
| Missing | 1 | For satisfaction |

^a^ Multiple response question.
